# Recent advances in the interaction of ferroptosis and immune-mediated inflammation in cardiovascular disease: mechanisms and therapeutic potential

**DOI:** 10.3389/fimmu.2025.1691705

**Published:** 2025-11-27

**Authors:** Zhihao Wang, Yexing Dang, Yuanyuan Li, Yu Zhang, Shuye Zhou, Zhiguo Zhang, Yuehui Wang

**Affiliations:** 1 Department of Geriatrics, The First Hospital of Jilin University, Changchun, China; 2 Department of Cardiovascular, The First Hospital of Jilin University, Changchun, China

**Keywords:** ferroptosis, immune-mediated inflammation, ferroptosis-immune-mediated inflammation interaction, cardiovascular system diseases, therapeutic targets

## Abstract

Ferroptosis is an iron-dependent form of programmed cell death primarily characterized by the inactivation of glutathione peroxidase 4 (GPX4), accumulation of lipid peroxides (LPO), and disruption of intracellular antioxidant defenses. Recent studies have revealed a close interplay between ferroptosis and immune-mediated inflammation, both of which contribute significantly to the pathogenesis of cardiovascular diseases (CVDs). In innate immunity, ferroptotic cells release damage-associated molecular patterns (DAMPs), such as high-mobility group box 1 (HMGB1), which activate the TLR–NF-κB signaling pathway, promote macrophage polarization toward the pro-inflammatory M1 phenotype, and induce the activation of NOD-like receptor protein 3 (NLRP3) inflammasomes, thereby amplifying inflammatory responses. In adaptive immunity, Th17 cells exacerbate cardiomyocyte ferroptosis by upregulating long-chain acyl-CoA synthetase 4 (ACSL4) via IL-17A secretion, whereas regulatory T cells protect by stabilizing GPX4 through IL-10. This review systematically delineates the intricate network linking ferroptosis and immune-mediated inflammation in CVDs, emphasizing the mechanisms by which ferroptosis modulates immune cell function, inflammatory cytokine release, and the oxidative stress. Moreover, we examined the involvement of this interaction in the pathophysiology of various CVDs, including atherosclerosis, myocardial infarction, myocardial ischemia–reperfusion injury (MIRI), heart failure, and cardiac arrhythmia. In addition, we provide a detailed analysis of the clinical translational potential of emerging therapeutic strategies targeting the ferroptosis–immune-inflammation axis, including interventions such as iron chelators, antioxidants, inflammation modulators, small-molecule inhibitors, and herbal compounds. By integrating the latest findings from basic and clinical research, this review offers novel insights and a theoretical framework for precision therapy in CVDs.

## 1 Introduction

Cardiovascular disease (CVD) is the leading cause of mortality and disability worldwide. According to the 2024 World Health Organization report, CVD accounts for 32.1% of all deaths worldwide, approximately 20.6 million annually, with ischemic heart disease and stroke comprising the major burden. Despite advances in contemporary management, including coronary revascularization and lipid-lowering therapies such as statins, two critical challenges remain unresolved. First, statin intolerance significantly limits therapeutic efficacy; nearly 20% of patients fail to achieve target low-density lipoprotein cholesterol levels or experience dose-limiting adverse effects, such as myalgia or hepatotoxicity. Second, the current anti-inflammatory strategies are suboptimal. The landmark CANTOS trial demonstrated that the IL-1β monoclonal antibody canakinumab reduced recurrent major adverse cardiovascular events in post-myocardial infarction (MI) patients (hazard ratio (HR): 0.85; 95% confidence interval (CI): 0.74–0.98; P = 0.021) (1); however, prohibitive costs and increased infection risk precluded its broad application. Similarly, NOD-like receptor protein 3 (NLRP3) inflammasome inhibitors (e.g., OLT1177) are undergoing phase II evaluation for systolic heart failure (HF) (2), underscoring the need for more precise immunomodulatory approaches. Collectively, these limitations highlight the incomplete understanding of CVD pathogenesis and the need to explore novel mechanisms of CVD.

Ferroptosis, a newly recognized form of programmed cell death, has emerged as a pivotal contributor to cardiovascular diseases (3). Defined by iron-dependent lipid peroxidation (LPO) and regulated by key molecules such as glutathione peroxidase 4 (GPX4) and the cystine/glutamate antiporter system Xc− (4), ferroptosis has been implicated in cardiomyocyte death during infarction and in multiple cardiovascular diseases (5). Studies investigating the role of ferroptosis in myocardial ischemia–reperfusion injury (MIRI) have primarily focused on the involvement of reactive oxygen species (ROS) (6), GPX4 (7), autophagy-dependent signaling (8), and endoplasmic reticulum stress (ERS) (9, 10). Ferroptosis has also been implicated in diverse cardiac disorders, including HF, Adriamycin-induced cardiomyopathy (11), diabetic cardiomyopathy (DCM) (12), sepsis-induced cardiac injury (13), and atrial fibrillation (14).

In parallel with these findings, immune-mediated inflammation has been increasingly recognized as a central driver of CVD. The NLRP3 inflammasome–IL-1β axis plays a particularly critical role in atherosclerosis (AS), where oxidized low-density lipoprotein (oxLDL) activates NLRP3, leading to IL-1β release, plaque instability, and thrombosis (15). Importantly, ferroptosis and inflammation form bidirectional regulatory circuits. Ferroptotic cells release damage-associated signals that promote macrophage polarization and cytokine release, while also shaping adaptive immune responses by modulating T cell function (16, 17). Conversely, ROS and pro-inflammatory cytokines, including TNF-α, IL-1β, and IL-6, sensitize cardiomyocytes and vascular endothelial cells to ferroptosis, perpetuating a vicious cycle (18).

Therefore, delineating the mechanisms underlying ferroptosis–immune crosstalk is critical for identifying new therapeutic targets. This review synthesizes current evidence from molecular pathways to translational applications, with the aim of advancing precision therapies for CVD.

## 2 Molecular interaction mechanisms of ferroptosis and immune inflammation

### 2.1 Overview of ferroptosis

Ferroptosis, first identified by Stockwell et al. in 2012, is a regulated form of cell death that is distinguished from apoptosis and necroptosis by its unique biochemical and morphological features (19). The hallmarks of this condition include aberrant iron accumulation, disrupted amino acid and lipid metabolism, and excessive LPO. Morphologically, ferroptotic cells display reduced or absent mitochondrial cristae, condensed membranes, and a lack of apoptotic bodies (20). These alterations underscore the mechanistic and pathological distinctions between ferroptosis and other modes of cell death.

Ferroptosis has since been implicated in ischemia–reperfusion injury, hepatic and renal fibrosis, neurodegenerative disorders such as Alzheimer’s disease, and cancer (6, 20–24). Within the cardiovascular system, ferroptosis contributes to the death of cardiomyocytes, endothelial cells, and smooth muscle cells, thereby influencing diverse cardiovascular diseases (25, 26). Mechanistically, three major drivers orchestrate ferroptosis:

1. Iron metabolism dysregulation: Elevated iron uptake through transferrin receptor 1 (TFR1) and divalent metal transporter 1 (DMT1), combined with ferritin degradation via autophagy, increases intracellular free iron levels, promoting hydroxyl radical generation through the Fenton reaction (27).
2. Antioxidant defense failure: The cystine/glutamate antiporter system Xc− (comprising SLC7A11/xCT and SLC3A2/4F2hc) imports cystine for glutathione (GSH) synthesis. GPX4 utilizes GSH to detoxify lipid peroxides (LPO), thereby preventing ferroptosis. Pharmacological inhibitors, such as erastin (targeting system Xc−) and RSL3 (inhibiting GPX4), strongly induce ferroptosis (28). Thus, the Xc−–GSH–GPX4 axis constitutes a central safeguard against ferroptosis.
3. Lipid metabolism reprogramming: Polyunsaturated fatty acid (PUFA)-containing phospholipids (e.g., PE-AA/AdA) undergo peroxidation via lipoxygenases (LOXs) and free radicals, leading to membrane rupture (29). Enzymes such as acyl-CoA synthetase acyl-CoA synthetase 4 (ACSL4) and lysophosphatidylcholine acyltransferase 3 (LPCAT3) regulate the biosynthesis of PUFA-enriched phospholipids, thereby determining cellular susceptibility to ferroptosis (30, 31).

Together, these mechanisms establish ferroptosis as a tightly regulated and pathologically relevant mode of cell death.

### 2.2 Immune-mediated inflammation

The immune system defends the host by eliminating pathogens and abnormal cells through both innate and adaptive mechanisms (32). Macrophages, dendritic cells (DCs), and natural killer (NK) cells play central roles in innate community. Macrophages recognize pathogen-associated molecular patterns (PAMPs) through pattern recognition receptors, which activate the NF-κB signaling pathway and stimulate the release of pro-inflammatory cytokines, including TNF-α and IL-6. Simultaneously, the assembly and activation of the NLRP3 inflammasome promote the maturation of IL-1β and IL-18, further amplifying inflammatory responses.

The cyclic GMP–AMP synthase–stimulator of interferon genes (cGAS–STING) pathway is a key mechanism that links cytosolic DNA sensing to innate immunity. Under autoimmune conditions, self-DNA released from damaged cells or neutrophil extracellular traps is detected by cGAS, which generates cGAMP to activate STING. Activated STING induces robust expression of type I interferons (IFN-α/β) and interferon-stimulated genes via the TBK1–IRF3 axis, forming the characteristic ‘type I interferon signature.’ Persistent type I interferon signaling not only serves as a hallmark of systemic lupus erythematosus but also disrupts immune tolerance, promotes autoantibody production, and enhances pathogenic T cell responses (e.g., Th17), ultimately driving chronic inflammation and tissue injury.

DCs, as professional antigen-presenting cells, capture antigens, undergo maturation, and migrate to lymphoid tissues, where they present antigens via major histocompatibility complex (MHC) molecules to activate naive T cells, thereby bridging the innate and adaptive immunity. NK cells contribute to the regulation of early inflammation in anti-infection and anti-tumor responses through cytotoxic activity and the secretion of cytokines, such as IFN-γ.

In adaptive immunity, the differentiation balance of CD4^+^ T-cells is critical for controlling inflammation. Th17 cells recruit neutrophils and exacerbate tissue inflammation through pro-inflammatory cytokines, including IL-17 and IL-22, whereas regulatory T (Treg) cells maintain immune tolerance by secreting inhibitory cytokines, such as IL-10 and TGF-β. Dysregulation of the Th17/Treg axis is a hallmark of chronic inflammation in various autoimmune diseases (33).

Inflammation, a core component of the immune response, manifests as redness, swelling, heat, and pain, serving to eliminate harmful stimuli and initiate tissue repair. During bacterial infection, immune cells, especially macrophages, activate NF-κB and NLRP3 inflammasome signaling upon pathogen recognition, releasing large amounts of pro-inflammatory cytokines and triggering acute inflammation. In autoimmune diseases, persistent autoantibody-mediated activation of DCs and macrophages disrupts the Th17/Treg balance, establishing a chronic inflammatory loop characterized by sustained activation of the NF-κB, NLRP3, and cGAS–STING pathways, ultimately leading to tissue damage and disease progression. Collectively, immune-mediated inflammation represents a tightly regulated network of cells and signaling pathways. Detailed elucidation of these cellular and molecular mechanisms provides new insights and potential therapeutic targets for modulating inflammatory responses.

Innate immunity plays a pivotal role in early cardiovascular injuries. Following myocardial ischemia or endothelial injury, damage-associated molecular patterns (DAMPs), such as high-mobility group box 1 (HMGB1) and ATP, activate macrophages and neutrophils via Toll-like receptors (TLRs) and the NLRP3 inflammasome (34). Macrophages, the central mediators of inflammation, exhibit phenotypic plasticity: M1 macrophages release pro-inflammatory cytokines (TNF-α, IL-6, and IL-1β), whereas M2 macrophages secrete anti-inflammatory mediators (IL-4 and IL-10). The balance between these subsets determines whether CVD progresses or regresses (35). For example, M1 macrophages destabilize atherosclerotic plaques (36), whereas IL-37 mitigates inflammation by suppressing macrophage ferroptosis via Nrf2 activation (37). Additionally, CRP, IL-6, and TNF-α, which are produced by innate immune cells, are strongly linked to MI, coronary artery disease, and stroke (38). Adaptive immunity also modulates CVD progression. CD4+ Th1 cells exacerbate AS through interferon-γ secretion, whereas Tregs confer protection. The role of B cells is context-dependent; some subsets produce protective antibodies that attenuate myocardial injury, whereas others facilitate atherogenesis (39, 40).

Immune inflammation drives CVD by linking innate and adaptive immune responses. Its interplay with ferroptosis forms a vicious cycle that aggravates tissue and cardiovascular injury.

### 2.3 Molecular pathways of ferroptosis triggering immune-mediated inflammatory response

#### 2.3.1 Release of DAMPs

Cardiomyocytes can release DAMPs in response to stress, hypertension, metabolic syndrome, ischemia–reperfusion injury, or other pathological stimuli. These DAMPs, including heat shock protein 60 (HSP60) and HMGB1, initiate sterile inflammatory responses by engaging pattern-recognition receptors on innate immune cells. Ferroptosis amplifies cellular immunogenicity by releasing DAMPs and proinflammatory mediators, thereby fostering a proinflammatory tissue microenvironment (41). Among these, HMGB1 is a prototypical DAMP (42). During ferroptosis, HMGB1 translocates from the nucleus to the cytoplasm and is released extracellularly, with its release levels correlating with the severity of ferroptosis. Mechanistically, HMGB1 binds to TLR4, activating the IKK complex through a MyD88-dependent pathway, which drives NF-κB nuclear translocation and the expression of pro-inflammatory cytokines (43). Consistent with this, Zhu et al. reported that HMGB1 released from ferroptotic cardiomyocytes robustly activates innate immune responses through the TLR4/NF-κB axis (44). Moreover, neutralization of HMGB1 with specific antibodies attenuates macrophage-mediated inflammatory responses induced by ferroptosis (45). In the MIRI model, inhibition of this central signaling axis significantly alleviates ferroptosis-related inflammatory damage and improves cardiac function. These findings establish the HMGB1–TLR4 signaling axis as a critical mediator of ferroptosis-associated immune inflammation, highlighting it as a potential therapeutic target for CVDs (46).

#### 2.3.2 Iron ion-mediated inflammation

Excess free iron promotes the generation of hydroxyl radicals through the Fenton reaction, resulting in direct cellular damage and activation of NLRP3 inflammasomes, thereby facilitating the maturation and secretion of IL-1β (47). As a potent proinflammatory cytokine, mature IL-1β orchestrates the activation and effector functions of multiple immune cell populations. In DCM, iron overload is positively correlated with the extent of cardiac inflammation (48), further implicating iron dysregulation in the inflammatory pathology of CVD.

#### 2.3.3 Lipid metabolism-mediated inflammation

Oxidized phospholipids (OxPLs) are critical mediators of macrophage polarization toward the pro-inflammatory M1 phenotype. A hallmark of ferroptosis is the rewiring of lipid metabolism, accompanied by excessive accumulation of LPO products. LPO oxidation is a central process in the metabolic pathways of ferroptosis and serves as a potential mechanism driving ferroptosis-associated inflammation. OxPLs modulate key cellular signaling pathways, thereby influencing cellular metabolism and inflammatory responses. Among these, oxidized phosphatidylethanolamine (OxPE) and oxidized phosphatidylcholine (OxPC) by ferroptotic cells are recognized by macrophages through scavenger receptors, including CD36 and TLR2. These OxPLs act as pivotal mediators, promoting macrophage polarization toward the pro-inflammatory M1 phenotype (49).

**Figure 1:**
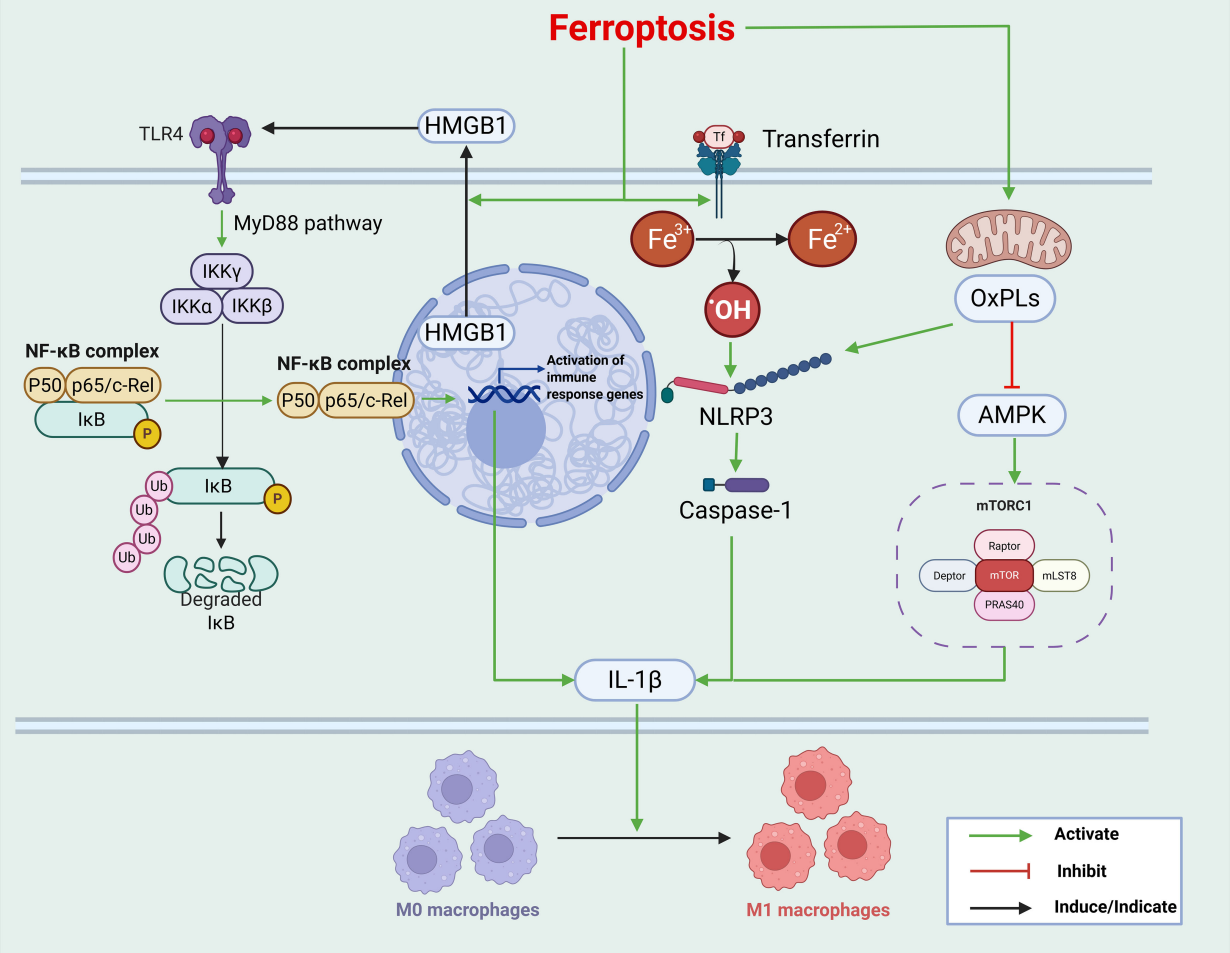
Ferroptosis activates immune-inflammatory responses through multiple mechanisms: 1. Release of DAMPs: HMGB1, a prototypical DAMP, translocates from the nucleus to the cytoplasm and is subsequently released into the extracellular space during ferroptosis. The binding of HMGB1 to TLR4 activates the IKK complex via the MyD88-dependent pathway, which in turn activates NF-κB. Activated NF-κB translocates to the nucleus, initiating the transcription of pro-inflammatory cytokines. 2. Iron ion–mediated inflammation: Free iron catalyzes the formation of hydroxyl radicals through the Fenton reaction, directly causing cellular damage and activating NLRP3 inflammasomes. This activation promotes the maturation and secretion of IL-1β in the macrophages. 3. OxPLs promote macrophage M1 polarization: OxPLs interact with inflammasome components to induce the assembly and activation of NLRP3 inflammasomes. Activated NLRP3 recruits and activates caspase-1, which cleaves pro–IL-1β into its mature form. In addition, OxPLs inhibit AMPK activity and enhance mTORC1 signaling, leading to the upregulation of pro-inflammatory cytokine expression. This figure was created using BioRender (biorender.com).

Two principal mechanisms have been proposed for OxPL-induced macrophage M1 polarization (50). 1) NLRP3 inflammasome activation: OxPLs facilitate the assembly and activation of NLRP3 inflammasomes by interacting with its components. This process is mediated by intracellular signaling events, including K^+^ efflux, Ca²^+^ flux, ROS accumulation, and lysosomal damage (51). Activated NLRP3 inflammasomes recruit and activate caspase-1, which cleaves pro–IL-1β into its mature form (52). Mature IL-1β subsequently drives macrophage differentiation from the M0 (unpolarized) state to the M1 phenotype (50). M1 macrophages exhibit enhanced pro-inflammatory potential, characterized by elevated secretion of cytokines and increased expression of M1-associated genes including CXCL10 (52, 53). 2) AMPK–mTORC1 signaling axis: Under oxidative stress, OxPLs inhibit AMPK activity, leading to the activation of the mTORC1 signaling pathway. This activation upregulates the expression of pro-inflammatory cytokines, including TNF-α and IL-6 (54), and promotes macrophage activation, thereby reinforcing an inflammatory microenvironment (**Figure 1**).

In summary, ferroptosis amplifies immune-mediated inflammation through multiple mechanisms, including the release of DAMPs, iron ion-dependent pathways, and alterations in lipid metabolism.

### 2.4 Feedback mechanisms of immune-mediated inflammation regulating ferroptosis

Immune-mediated inflammation arises from a complex network of signaling pathways and molecular interactions (55–57). Mounting evidence indicates that dysregulated inflammatory responses critically disrupt iron metabolism and perturb the cellular redox homeostasis. Pro-inflammatory cytokines modulate ferritin synthesis, exerting direct effects on intracellular and tissue-level iron storage (58). Next, we elaborate on the relationship between immune cells, inflammatory cytokines, and ferroptosis. The roles of relevant pathways, including NF-κB, inflammasome, and JAK-STAT signaling, in ferroptosis are discussed in subsequent sections.

#### 2.4.1 Macrophage polarization

M1-type macrophages promote ferroptosis during inflammatory responses through multiple mechanisms: 1) Cytokine secretion: M1 macrophages secrete pro-inflammatory cytokines that activate intracellular oxidative stress responses and increase LPO accumulation, thereby facilitating ferroptosis (16). 2) ROS generation and antioxidant suppression: M1 macrophages release ROS, which deplete GSH and inhibit GPX4 activity, thereby disrupting the cellular antioxidant defense system (16, 59). In addition, TNF-α produced by M1 macrophages suppresses the activity of the transcription factor Nrf2, reducing GSH synthesis and weakening oxidative defenses, ultimately inducing ferroptosis (60). 3) Disruption of iron homeostasis: M1 macrophages contribute to intracellular iron overload by upregulating TFR1 and downregulating ferroportin, thereby promoting free iron accumulation and amplifying the Fenton reaction (60, 61). Furthermore, M1 macrophages can activate ACSL4, increasing cellular sensitivity to LPO and promoting ferroptosis in tumor cells (62).

In contrast, M2 macrophages are anti-inflammatory and resistant to ferroptosis. They secrete IL-4 and IL-13, which upregulate the cystine/glutamate antiporter subunit SLC7A11, thereby enhancing cellular defense against ferroptosis (63). Clinically, in valvular atrial fibrillation (VAF), an imbalance in the M1/M2 macrophage ratio is closely associated with the severity of cardiomyocyte ferroptosis (64).

#### 2.4.2 T-cell subpopulation-mediated regulation

The adaptive immune system, particularly distinct T cell subpopulations, plays a pivotal role in regulating ferroptosis. By secreting specific cytokines, different T-cell subsets shape a complex regulatory network that influences iron metabolism and ferroptotic signaling.

Th1 cells promote ferroptosis: Th1 cells promote ferroptosis primarily through the secretion of IFN-γ. IFN-γ inhibits the expression of SLC7A11 by activating the JAK–STAT pathway. SLC7A11, a key component of system Xc^-^ responsible for cystine uptake and glutamate export, is essential for GSH synthesis; its inhibition lowers intracellular cystine levels and impairs GSH production. This reduction in GSH diminishes GPX4 activity and hinders the clearance of LPOs, ultimately driving ferroptosis (65, 66).

Tregs cells inhibit ferroptosis: In contrast, Tregs exert anti-ferroptotic effects by stabilizing GPX4 and suppressing LPO through the action of IL-10, via two complementary mechanisms: 1) Direct upregulation of GPX4 through the IL-10R–STAT3 axis: Binding of IL-10 to its receptor induces STAT3 phosphorylation, which enhances GPX4 transcription and effectively prevents ferroptosis (67). 2) Inhibition of ALOX15 via suppression of pro-inflammatory signals: Through STAT3 activation, IL-10 inhibits TNF-α/NF-κB signaling, leading to the downregulation of its downstream target ALOX15, a key lipoxygenase in LPO, and thereby blocking ferroptosis (68, 69).

#### 2.4.3 Inflammasome activation

**Figure 2:**
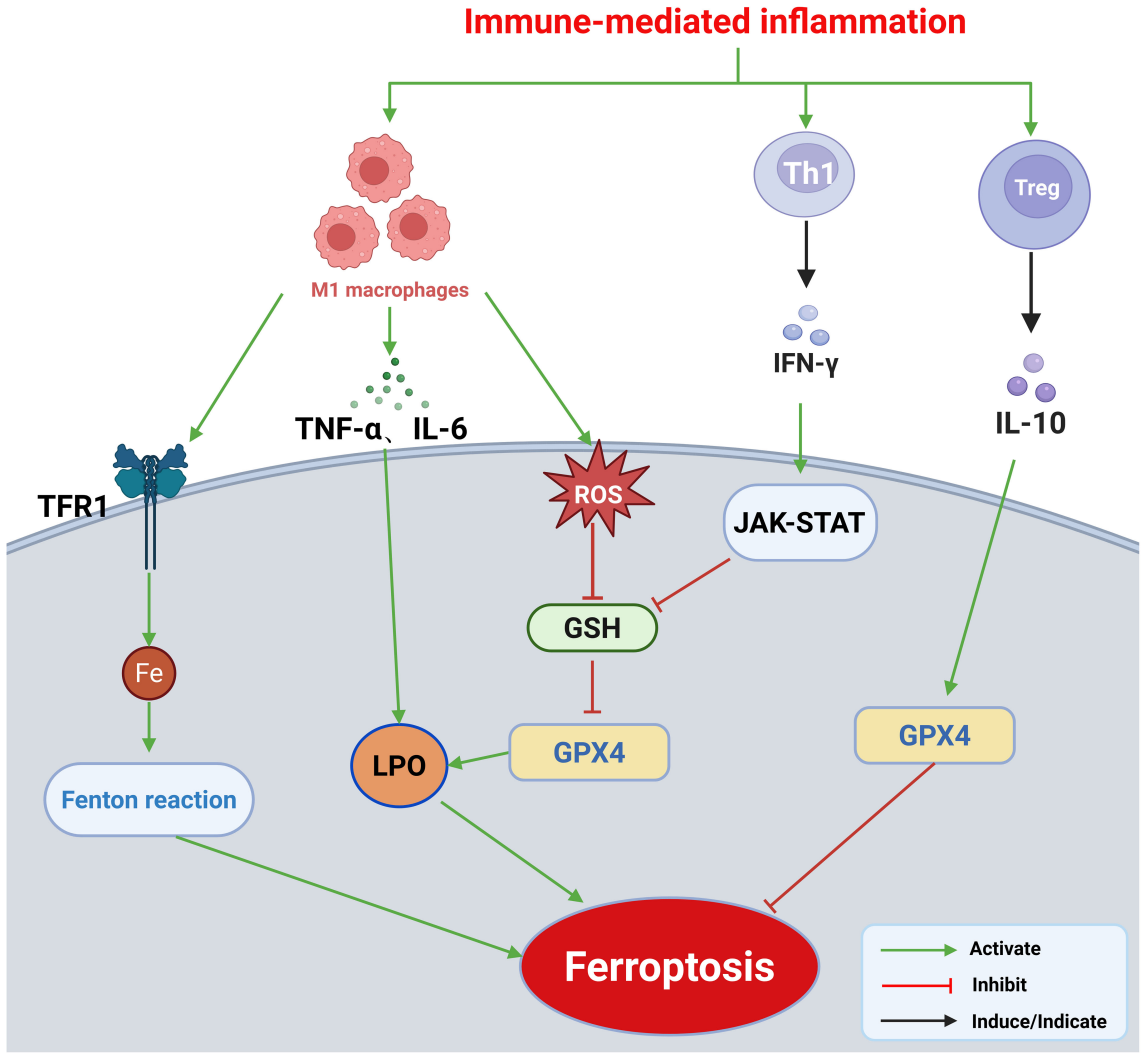
Mechanisms by which immune-mediated inflammation regulates ferroptosis: 1. Macrophage polarization: M1 macrophages release pro-inflammatory cytokines, including TNF-α and IL-6, which enhance intracellular oxidative stress and increase LPO accumulation, thereby promoting ferroptosis in macrophages. M1 macrophages also produce ROS, further depleting intracellular GSH and inhibiting GPX4 activity, thereby compromising the cellular antioxidant defense system. Additionally, M1 macrophages disrupt iron homeostasis by upregulating TFR1 and downregulating ferroportin, leading to intracellular free iron overload and amplification of the Fenton reaction. 2. T cell subpopulation–mediated regulation: IFN-γ secreted by Th1 cells inhibits ferroptosis via activation of the JAK–STAT signaling pathway. In contrast, Tregs maintain GPX4 stability and suppress ferroptosis through IL-10, which exerts its effects via multiple pathways. This figure was created using BioRender (biorender.com).

NLRP3 inflammasome assembly not only induces IL-1β release but also amplifies ferroptosis through NEK7 (NIMA-related kinase 7)-mediated mitochondrial injury (70). As previously described, activation of the NLRP3 inflammasome triggers caspase-1, which mediates the maturation and release of IL-1β and IL-18. Caspase-1 also cleaves gasdermin D (GSDMD), initiating pyroptosis, an inflammatory form of programmed cell death (71). NEK7 serves as a critical regulator of NLRP3 activation by directly binding to the leucine-rich repeat (LRR) domain of NLRP3, inducing a conformational transformation into a disc-like structure. This enables the recruitment of the adaptor protein ASC, thereby initiating inflammasome activation (72). NEK7 may also promote NLRP3 activation by sensing mitochondrial ROS (mtROS) (73). Mitochondrial injury accelerates ferroptosis by increasing intracellular iron accumulation and LPO production. In a liver ischemia–reperfusion model, inhibition of the caspase-6–RIPK1–IκBα axis suppressed NLRP3 activation and hepatocyte ferroptosis (74). Given the parallels between hepatic and cardiac injuries, this pathway may be particularly relevant in the context of myocardial damage (**Figure 2**).

**Figure 3:**
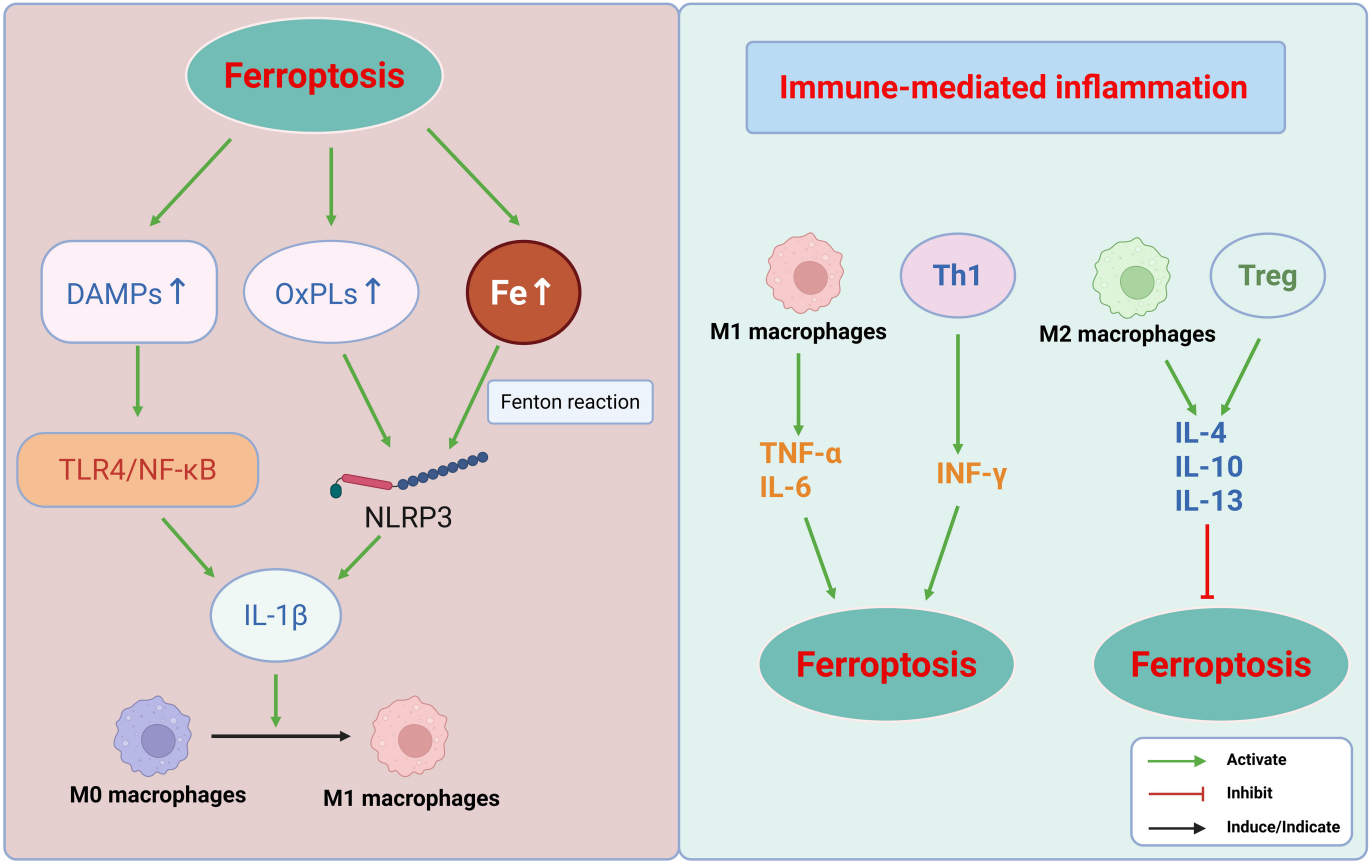
Schematic illustrating the bidirectional interplay between ferroptosis and immune-mediated inflammation. This figure depicts a self-amplifying loop underlying inflammatory tissue injury. Left: Ferroptosis promotes immune responses. Ferroptosis releases damage-associated molecular patterns (DAMPs), lipid peroxidation products (e.g., OxPLs), and free iron, which activate immune pathways such as TLR4/NF-κB and the NLRP3 inflammasome, promoting M1 macrophage polarization. Right: Immunity regulates ferroptosis. The ensuing immune response bidirectionally modulates the ferroptosis. Pro-inflammatory components (M1 macrophages and Th1 cells) exacerbate ferroptosis by inducing oxidative stress and iron overload, whereas anti-inflammatory components (M2 macrophages and Tregs) suppress ferroptosis by enhancing the antioxidant defenses. This reciprocal crosstalk forms a feed-forward loop that amplifies the cellular injury and inflammation. This figure was created using BioRender (biorender.com).

In summary, immune-mediated inflammation facilitates ferroptosis through multiple mechanisms, including macrophage polarization, T cell subset modulation, and inflammasome activation. These insights offer a novel conceptual framework for understanding the intricate interplay between inflammation and ferroptosis (**Figure 3**).

## 3 Common regulatory nodes and key signaling pathways

In certain diseases, ferroptosis and immune-mediated inflammation intersect at multiple critical regulatory nodes and signaling pathways, as described in the following sections.

### 3.1 Nrf2–HO-1 axis

Nrf2, a key antioxidant transcription factor, upregulates genes such as HO-1, SLC7A11, and ferritin heavy chain 1 (FTH1), protecting cells from oxidative stress and inflammatory damage. Nrf2 activators, such as resveratrol, have demonstrated dual protective effects in models of AS and myocardial ischemia (75). Beyond its antioxidant role, Nrf2 participates in other critical pathways, including lipid metabolism, iron homeostasis, and energy metabolism, which are tightly regulated to modulate the ferroptosis. Yang et al. reported that polyphyllin I activates the Nrf2–HO-1–GPX4 axis, causing mitochondrial dysfunction and promoting ferroptosis to suppress hepatocellular carcinoma (HCC) progression (76). Xiong demonstrated that IL-27 regulates macrophage ferroptosis by suppressing the Nrf2–HO-1 signaling pathway in sepsis-induced acute respiratory distress syndrome (77). Liu et al. showed that ginsenosides regulate ferroptosis via the Nrf2–HO-1 and PI3K–AKT–mTOR pathways, reducing ferroptosis in a mouse model of iron-overloaded aplastic anemia and in Meg-01 megakaryocyte cells *in vitro* (78). Additionally, neutral polysaccharides from *Gastrodia elata* attenuated cerebral ischemia–reperfusion injury by inhibiting ferroptosis-mediated neuroinflammation via the Nrf2/HO-1 pathway (79). Moreover, 6-gingerol mitigated ferroptosis and inflammation in DCM by activating the Nrf2–HO-1 axis (80). Collectively, these studies highlight the Nrf2/HO-1 axis as a central regulator that coordinates antioxidant, anti-inflammatory, and anti-ferroptotic responses. Targeted activation of this pathway mitigates disease progression across diverse models, from cancer to ischemia–reperfusion injury, underscoring its broad therapeutic potential.

### 3.2 ACSL4–LPCAT3–ALOX15 axis

The ACSL4–LPCAT3–ALOX15 axis is as a central hub for the interplay between ferroptosis and inflammation. ACSL4 catalyzes the activation of PUFAs, such as arachidonic acid (AA) and adrenic acid (AdA), into acyl-CoA derivatives, including AA-CoA and AdA-CoA (81). LPCAT3 subsequently incorporates these activated fatty acids into membrane phospholipids, generating key lipid substrates for ferroptosis (82, 83). ALOX15 selectively oxidizes these phospholipids to produce peroxides, such as PE-AA-OOH and PE-AdA-OOH, inducing membrane damage and triggering ferroptosis (84, 85). The ACSL4–LPCAT3–ALOX15 axis amplifies inflammatory signaling. ACSL4-derived AA serves as a precursor for prostaglandins and leukotrienes, ALOX15 products such as 4-hydroxynonenal (4-HNE) activate the NLRP3 inflammasome, and LPCAT3 modulates TLR4 signaling by regulating membrane phospholipid composition. In multiple inflammatory models, small-molecule inhibitors targeting this axis such as the ACSL4 inhibitor thiazolidinediones and the ALOX15 inhibitor PD146176, simultaneously suppress ferroptosis and alleviate inflammation (86). In summary, this signaling axis functions as a central node that synergistically regulates both ferroptosis and inflammatory signal amplification by sequentially catalyzing the activation, esterification, and peroxidation of PUFAs, thereby providing promising therapeutic targets for related diseases.

### 3.3 NF-κB pathway

The canonical NF-κB pathway is central to inflammation and innate immunity (87–89) and engages in complex crosstalk with ferroptosis. NF-κB activation upregulates TFR1 and downregulates FTH1, promoting ferroptosis. In contrast, LPO induced by ferroptosis activates NF-κB, triggering inflammation and establishing a positive feedback loop. Under inflammatory stimuli, IκBα is phosphorylated and degraded, allowing NF-κB dimers to translocate into the nucleus and regulate downstream gene transcription. NF-κB suppresses the transcription of antioxidant genes, including GPX4, Quinone Oxidoreductase 1(NQO1), and heme oxygenase 1(HMOX1), exacerbating oxidative stress and ferroptosis. Moreover, NF-κB regulates extracellular iron utilization by modulating lipocalin 2 (LCN2) secretion. Considerable evidence supports the involvement of NF-κB signaling in ferroptosis (90). For instance, ubiquitin-specific protease 24 (USP24)-mediated NF-κB upregulation aggravates ferroptosis in DCM (91). Dimethyl fumarate (DMF) attenuates neuroinflammation and ferroptosis, improving cognitive dysfunction in rats with chronic cerebral hypoperfusion induced by double vessel occlusion via modulation of NF-κB signaling (92). Ferrostatin-1 (Fer-1), a ferroptosis inhibitor, reduced LPS-induced cardiac inflammation in a rat model of cardiac insufficiency by blocking the TLR4–NF-κB pathway, thereby improving cardiac function and survival (93). Collectively, these findings highlight the critical role of NF-κB–mediated inflammatory responses in regulating ferroptosis.

### 3.4 JAK–STAT pathway

The JAK–STAT signaling pathway is a major cytokine-regulated cascade essential for initiating innate immunity, coordinating adaptive responses, and modulating inflammation (94). IFN-γ is a pivotal cytokine that activates the JAK–STAT1 pathway, inducing the expression of interferon-stimulated genes (95), and participates in immune surveillance and inflammation regulation. IFN-γ enhances tumor cell sensitivity to ferroptosis inducers via the JAK–STAT pathway (96, 97). Specifically, IFN-γ inhibits the expression of SLC3A2 and SLC7A11 through the JAK–STAT–IRF1 axis, thereby promoting ferroptosis in HCC cells (97). Members of the STAT family also play key roles in ferroptotic processes. Activated STAT1 can inhibit System Xc^-^-driven ferroptosis (90), whereas STAT3 promotes intracellular iron accumulation by upregulating hepcidin and directly regulating ACSL4 transcription, collectively enhancing cellular susceptibility to ferroptosis (21, 98, 99). Thus, the JAK–STAT pathway is a critical functional link between immune regulation and ferroptosis. Recent studies have demonstrated that the anti-rheumatoid arthritis drug genoprofen upregulates iron-regulatory hormones via the classical JAK2–STAT3 pathway in human hepatocytes and mouse models (100). Moreover, JAK inhibitors exhibit dual efficacy in suppressing ferroptosis and alleviating cardiac inflammation in DCM models (27, 101). In summary, the JAK–STAT pathway functions as a bidirectional regulatory hub connecting immune responses and ferroptosis by controlling the key genes involved in iron metabolism and LPO. Targeted modulation of this pathway offers therapeutic potential for inhibiting inflammation and intervening in ferroptosis-related diseases.

### 3.5 cGAS–STING pathway

The cGAS–STING pathway is a key innate immune sensor that detects cytoplasmic DNA and triggers type I interferon and inflammatory responses in the host. It plays a critical role in DNA damage surveillance and antiviral defense. During ferroptosis, intracellular DNA damage or abnormal DNA accumulation can activate the cGAS–STING pathway, triggering downstream cascades such as NF-κB, which promotes the production of inflammatory factors and exacerbates ferroptosis (90). Naringenin has been reported to modulate the cGAS–STING pathway, alleviating mitochondrial dysfunction and ferroptosis in MIRI models (102).

### 3.6 MAPK pathway

**Figure 4:**
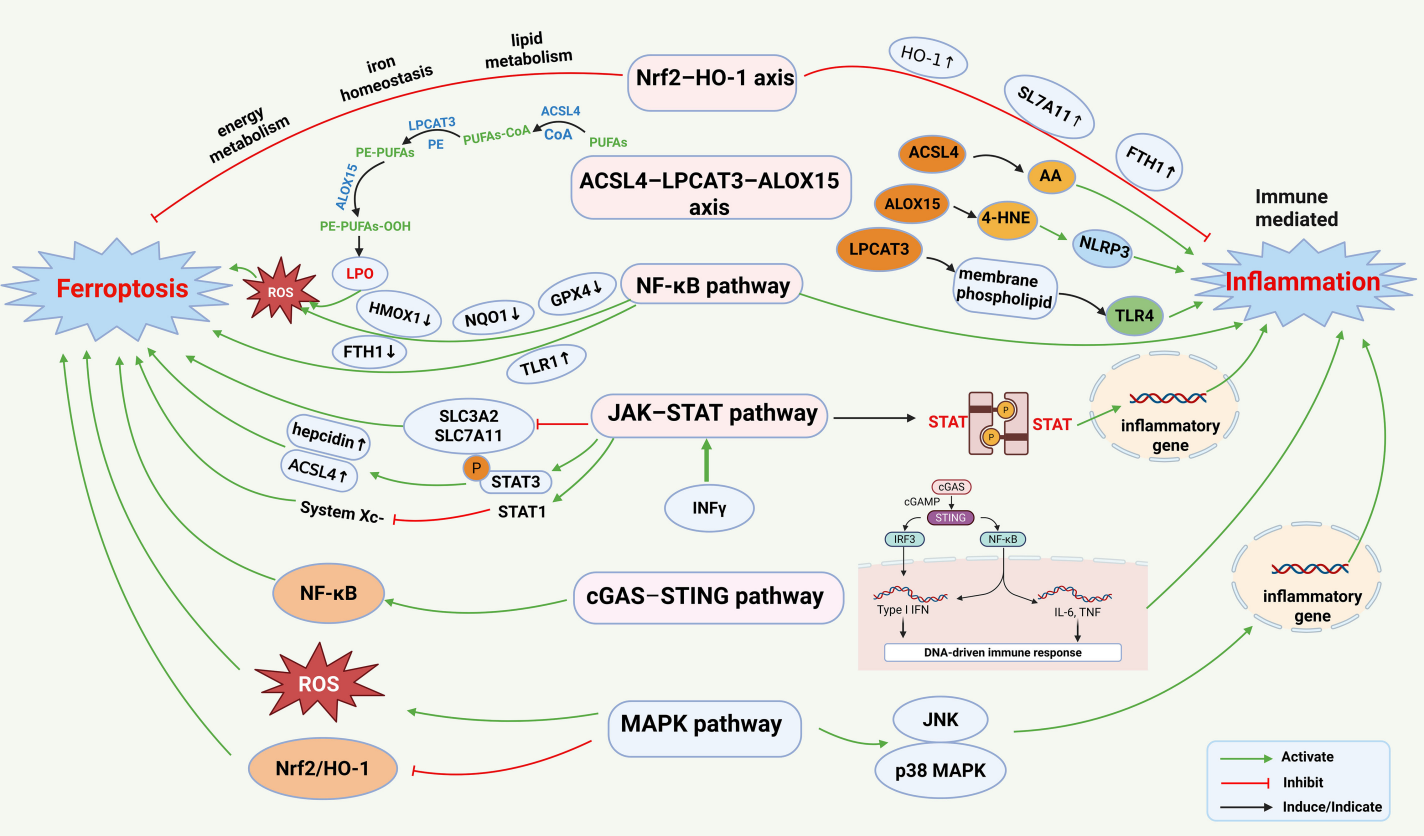
Ferroptosis and immune-mediated inflammation share several key signaling pathways: 1. Nrf2–HO-1 axis: Nrf2 upregulates the expression of genes such as HO-1, SLC7A11, and FTH1, providing protection against oxidative stress and inflammation. Nrf2 also participates in lipid metabolism, iron homeostasis, and energy metabolism, thereby regulating ferroptosis. 2. ACSL4–LPCAT3–ALOX15 axis: ACSL4 catalyzes the conjugation of intracellular PUFAs with CoA to form acyl-CoA derivatives. LPCAT3 transfers PUFA–CoA to lyso-phosphatidylethanolamine (lyso-PE), generating PE–PUFA. ALOX15 selectively oxidizes PE–PUFA to produce peroxidized derivatives. The accumulation of these peroxidized phospholipids compromises membrane integrity, ultimately triggering ferroptosis. In addition, ACSL4, ALOX15, and LPCAT3 independently contribute to inflammation via arachidonic acid (AA), 4-hydroxynonenal (4-HNE), and TLR4 signaling. 3. NF-κB pathway: NF-κB activation upregulates TFR1 and downregulates ferritin heavy chain (FTH1), promoting ferroptosis. LPO generated during ferroptosis can activate NF-κB, triggering inflammation and establishing a positive feedback loop. 4. JAK–STAT signaling: Engagement of the IFN-γ receptor activates the JAK–STAT1 pathway, which induces downstream genes and proteins that promote both ferroptosis and inflammation. 5. cGAS–STING pathway: Intracellular DNA damage or aberrant DNA accumulation activates the cGAS–STING pathway, triggering downstream cascades, such as NF-κB, thereby promoting inflammatory factor production and exacerbating ferroptosis. 6. MAPK pathway: Inflammatory cytokines activate MAPK signaling, disrupting intracellular redox homeostasis and iron metabolism, thus influencing ferroptosis. Concurrently, MAPK activation enhances the production of proinflammatory factors, amplifying the inflammatory response. This figure was created using BioRender (biorender.com).

The mitogen-activated protein kinase (MAPK) pathway encompasses key signaling cascades, including ERK, JNK, and p38, which are essential for cellular responses to stress, inflammation, and apoptosis. Inflammatory cytokines can activate MAPK signaling, disrupting intracellular redox homeostasis and iron metabolism, thereby influencing ferroptosis in the body. Concurrently, MAPK activation can further enhance the production of pro-inflammatory factors, amplifying the inflammatory response (103). Dapagliflozin attenuates MIRI by reducing ferroptosis via the inhibition of MAPK signaling (104). Additionally, CTRP12 ameliorates HF following MI by modulating the TAK1–p38 MAPK/JNK pathway, thereby mitigating apoptosis, oxidative stress, and inflammation (105) (**Figure 4**). In summary, the MAPK pathway establishes a vicious cycle between inflammation and ferroptosis, driving disease progression and representing a highly promising therapeutic target for intervention. More broadly, the core network governing ferroptosis–immune inflammation crosstalk is centered on key hubs, including Nrf2/HO-1 and ACSL4, and is mediated through bridging pathways such as NF-κB, JAK–STAT, cGAS–STING, and MAPK. Collectively, these nodes orchestrate redox homeostasis, iron metabolism, and LPO, thereby coupling the amplification of inflammatory responses with ferroptosis. This framework provides a compelling basis for the development of combinatorial therapeutic strategies for diseases such as cancer and cardiovascular disorders.

## 4 Interaction of ferroptosis and immune-mediated inflammation in CVDs

### 4.1 Atherosclerosis

Coronary atherosclerotic heart disease (CHD) is one of the most prevalent CVDs and commonly manifests as angina pectoris and MI. AS is the pathological basis of CHD and is recognized as a chronic inflammatory disorder in which ferroptosis and immune-inflammatory responses dynamically interact throughout disease progression.

Plaque formation stage: AS promotes arterial wall thickening and stiffening due to the accumulation of extracellular matrix, cholesterol, and cellular debris. Lipid metabolism plays a pivotal role in the development of plaques. OxLDL induces ferroptosis in vascular endothelial cells by upregulating TFR1 and acyl-CoA synthetase ACSL4, thereby exacerbating endothelial injury (106). Simultaneously, oxLDL activates NLRP3 inflammasomes in macrophages, amplifying local inflammation and ferroptotic cell death (34), which further aggravates AS (107).

Plaque progression stage: During plaque progression, macrophages acquire excess iron through erythrophagocytosis and subsequently undergo ferroptosis, releasing cholesterol crystals and oxPLs, thus establishing a vicious cycle (16). Macrophages in unstable plaques exhibit pronounced ferroptotic characteristics that correlate positively with the risk of plaque rupture (108). Heat shock protein B1 (HSPB1) inhibits oxLDL-induced ferroptosis in vascular smooth muscle cells (VSMCs) by suppressing dipeptidyl peptidase 4 (DPP4) via NF-κB, modulating the interplay between inflammation and ferroptosis, and presenting a potential therapeutic strategy for AS (109). Additionally, the metabolite Neu5Ac promotes the degradation of SLC3A2, inducing ferroptosis in vascular endothelial cells and accelerating AS progression in ApoE^−/−^ mice. Concurrently, elevated IL-1β and ICAM-1 expression enhance monocyte adhesion to the endothelium. Notably, the ferroptosis inhibitor, Fer-1, can reverse these effects (110). Moreover, IL-23p19 deficiency or Fer-1 treatment improves cardiac remodeling and function by inhibiting M1 macrophage polarization and ferroptosis (111).

In summary, ferroptosis and immune-mediated inflammation synergistically contribute to AS and CVD pathogenesis by targeting macrophages, endothelial cells, and VSMCs. Consequently, anti-ferroptosis interventions, such as HSPB1 modulation and Fer-1 administration, represent promising therapeutic strategies.

### 4.2 Myocardial infarction

MI is a life-threatening condition caused by acute coronary artery obstruction, resulting in insufficient blood supply to the myocardium and subsequent necrosis. Accumulating evidence indicates that ferroptosis plays a critical role in this pathway. Under hypoxic or ischemic conditions, GPX4 activity is suppressed, whereas HIF-1α upregulates the iron transporter TFR1. These alterations collectively promote LPO accumulation and intracellular iron overload, thereby inducing ferroptosis in cardiomyocytes (112–114). Ferroptosis not only directly mediates cardiomyocyte death but also triggers secondary inflammatory responses, exacerbating cardiac dysfunction and adverse remodeling. Accordingly, the inhibition of ferroptosis has emerged as a potential therapeutic strategy for preserving cardiac function following MI (115). Notably, Sestrin2, upregulated in MI patients with anxiety and depression, provides protection against inflammation, oxidative stress, and ferroptosis in experimental models via activation of the LKB1/AMPK signaling pathway (116).

### 4.3 Myocardial ischemia–reperfusion injury

In patients with MI, percutaneous coronary intervention (PCI) restores blood flow; however, reperfusion can paradoxically exacerbate myocardial injury, a phenomenon known as MIRI. MIRI represents a classic pathological process in which ferroptosis closely interacts with immune-mediated inflammation.

Although blood flow is restored, reperfusion triggers a massive burst of ROS in cardiomyocytes, initiating ferroptosis (117). Furthermore, DAMPs, such as HMGB1 and ATP, released from injured cells activate innate immune responses. This activation promotes IL-17 production and neutrophil recruitment, thereby exacerbating cardiac injury (118, 119). Evidence indicates a reciprocal, self-propagating cycle between ferroptosis and inflammation. Initial ferroptotic cell death activates NF-κB and JNK signaling, leading to the production of inflammatory factors that feedback to intensify ERS and further drive ferroptosis, progressively amplifying tissue injury (117). For instance, NF-κB activation exacerbates myocardial damage by increasing ROS levels and promoting ferritin degradation (120). Similarly, JNK pathway activation contributes significantly to propagating the inflammatory response initiated by ferroptosis (120). Importantly, pharmacological interventions, such as glycyrrhizin, can simultaneously suppress ferroptosis and inflammation through the HMGB1-TLR4-GPX4 axis, highlighting this pathway as a promising therapeutic target (44).

### 4.4 Heart failure

HF encompasses clinical syndromes arising from structural or functional cardiac alterations, and is characterized by impaired ventricular filling, reduced ejection capacity, and insufficient cardiac output to meet tissue metabolic demands. This results in pulmonary and/or systemic congestion and inadequate organ perfusion. HF represents the terminal stage of diverse cardiovascular disorders and involves low-grade immune-mediated inflammation (121). In cardiomyocytes, iron accumulation triggers ferroptosis and the release of DAMPs, such as HMGB1, which promotes immune cell infiltration. These immune cells exacerbate injury by secreting pro-inflammatory factors and releasing additional iron, thereby sustaining a cycle of oxidative stress and ferroptosis that drives myocardial fibrosis, ventricular remodeling, and HF progression (122, 123).

Pressure overload-induced myocardial hypertrophy is a common etiology of HF. Mechanical stress from pressure overload, such as aortic constriction or hypertension, induces intracellular iron accumulation and ferroptosis in cardiomyocytes, establishing a self-perpetuating cycle of cardiomyocyte death. Moreover, myocardial iron overload contributes to cardiomyocyte loss, exacerbates fibrosis, and impairs the cardiac function. Lipidomic and RNA sequencing analyses by Wang et al. revealed elevated phosphatidylethanolamine (PE) and Acsl4 expression in mice with TAC-induced HF. Overexpression of Acsl4 in cardiomyocytes amplified ferroptosis-induced pressure overload dysfunction. Mechanistically, Acsl4-dependent ferroptosis activates inflammatory necroptosis pathways, increasing IL-1β production, and IL-1β neutralization post-TAC improves cardiac function in Acsl4 transgenic mice (124). In a rat HF model, puerarin inhibited ferroptosis and protected against pressure overload-induced cardiomyocyte injury (125). GPX4 downregulation is strongly associated with cardiomyocyte ferroptosis and has important implications for HF pathology. Researchers using single-cell sequencing identified “ferroptosis-susceptible” cardiomyocytes with low GPX4 expression in the failing heart, which exhibited heightened susceptibility to ferroptosis (126). The IL-6–STAT3 pathway also contributes to inflammation and iron homeostasis, which may lead to endothelial injury and iron overload. Elabela, an endogenous peptide hormone, attenuates iron-induced ferroptosis, myocardial remodeling, fibrosis, and cardiac dysfunction in hypertensive mice via IL-6–STAT3–GPX4 modulation (127).

At the same time, dysregulated lipid metabolism and iron homeostasis synergistically drive ferroptosis, causing cardiomyocyte injury and dysfunction (128). In HF, particularly in diabetes or obesity, myocardial fatty acid oxidation is elevated, resulting in LPO accumulation and providing a substrate for ferroptosis (129). The iron-regulated protein (IRP)–iron-responsive element (IRE) system maintains intracellular iron homeostasis. Oxidative stress and hypoxia can disrupt IRP activity, creating an iron imbalance that further promotes ferroptosis (130). Collectively, these mechanisms contribute to cardiomyocyte injury and progressive cardiac dysfunction.

In summary, the “ferroptosis–inflammation” vicious cycle represents a critical pathological mechanism underlying HF. This cycle is initiated by iron overload and LPO, amplified by key regulators such as ACSL4 and GPX4, and reinforced by inflammatory signals, including IL-1β and STAT3, collectively driving myocardial remodeling and functional decline.

### 4.5 Other CVDs

#### 4.5.1 Atrial fibrillation

Atrial fibrillation (AF) is a common cardiovascular disorder in the elderly. Atrial tissue from patients with valvular AF (VAF) showed substantial iron accumulation and GPX4 downregulation, which correlated positively with inflammatory infiltration. Bioinformatics analyses have revealed a significant association between ferroptosis-related genes and immune infiltration in VAF (131). Jin et al. investigated the relationship between AF, ferroptosis, and inflammation (132). Yu et al. reported significant iron accumulation and GPX4 downregulation in the left atrial tissues of AF patients, associated with myocardial fibrosis (133). GPX4 expression is also positively correlated with inflammatory markers, including TNF-α and IL-1β (134).

#### 4.5.2 Diabetic cardiomyopathy

DCM is a diabetes-associated cardiac complication that initially presents with left ventricular hypertrophy and diastolic dysfunction and later progresses to HF, characterized by systolic impairment. Its pathogenesis involves multiple mechanisms, including inflammation, advanced glycation end products (AGEs), angiotensin II, and ferroptosis, all of which contribute to cardiomyocyte injury and functional decline. Hyperglycemia drives LPO accumulation in cardiomyocytes and shifts energy metabolism from glycogenolysis to fatty acid oxidation, thereby enhancing lipotoxicity and triggering ferroptosis. The suppression of Nrf2 activity and GPX4 expression further weakens the cellular defense against ferroptotic stress. Ferroptosis releases ROS and DAMPs, which reinforce oxidative injury and sustain a self-perpetuating cycle that accelerates DCM progression. Under hyperglycemic conditions, AGEs promote ferroptosis in cardiomyocytes and activate macrophage inflammation through RAGE signaling, amplifying this deleterious feedback loop (135). In a mouse model of diabetic myocardial injury, FACL4 expression and iron accumulation were elevated, whereas GPX4 levels declined. Canagliflozin attenuates inflammation and ferroptosis by activating AMPK, thereby reducing cardiomyocyte lipotoxicity in DCM (136). Clinically, serum ferroptosis biomarkers, such as MDA, correlate with the severity of diastolic dysfunction in diabetic patients (137), underscoring the pivotal contribution of ferroptosis and inflammation to DCM pathogenesis (80).

#### 4.5.3 Aortic aneurysms and dissection

Aortic aneurysms and coarctation are severe cardiovascular disorders characterized by inflammation and VSMC injury. Research indicates that ferroptosis is a key mechanism underlying vascular smooth muscle cell (VSMC) death and subsequent degeneration of the aortic media. In ascending thoracic aortic aneurysms/acute Stanford type A aortic dissection (ATAA/ATAAD) and abdominal aortic aneurysms (AAA), VSMC ferroptosis directly compromises the structural integrity of the vascular wall (138). Concurrently, ferroptosis activates inflammatory responses, induces cytokine release, and upregulates matrix metalloproteinase-9 (MMP-9), which degrades the extracellular matrix, collectively exacerbating arterial wall destruction (139). Moreover, imbalances in iron metabolism, whether due to cellular dysfunction from iron deficiency (140) or iron-induced cell death caused by iron overload (141), disrupt VSMC homeostasis, ultimately promoting aortic intima-media thickening and structural damage to the arterial wall.

**Figure 5:**
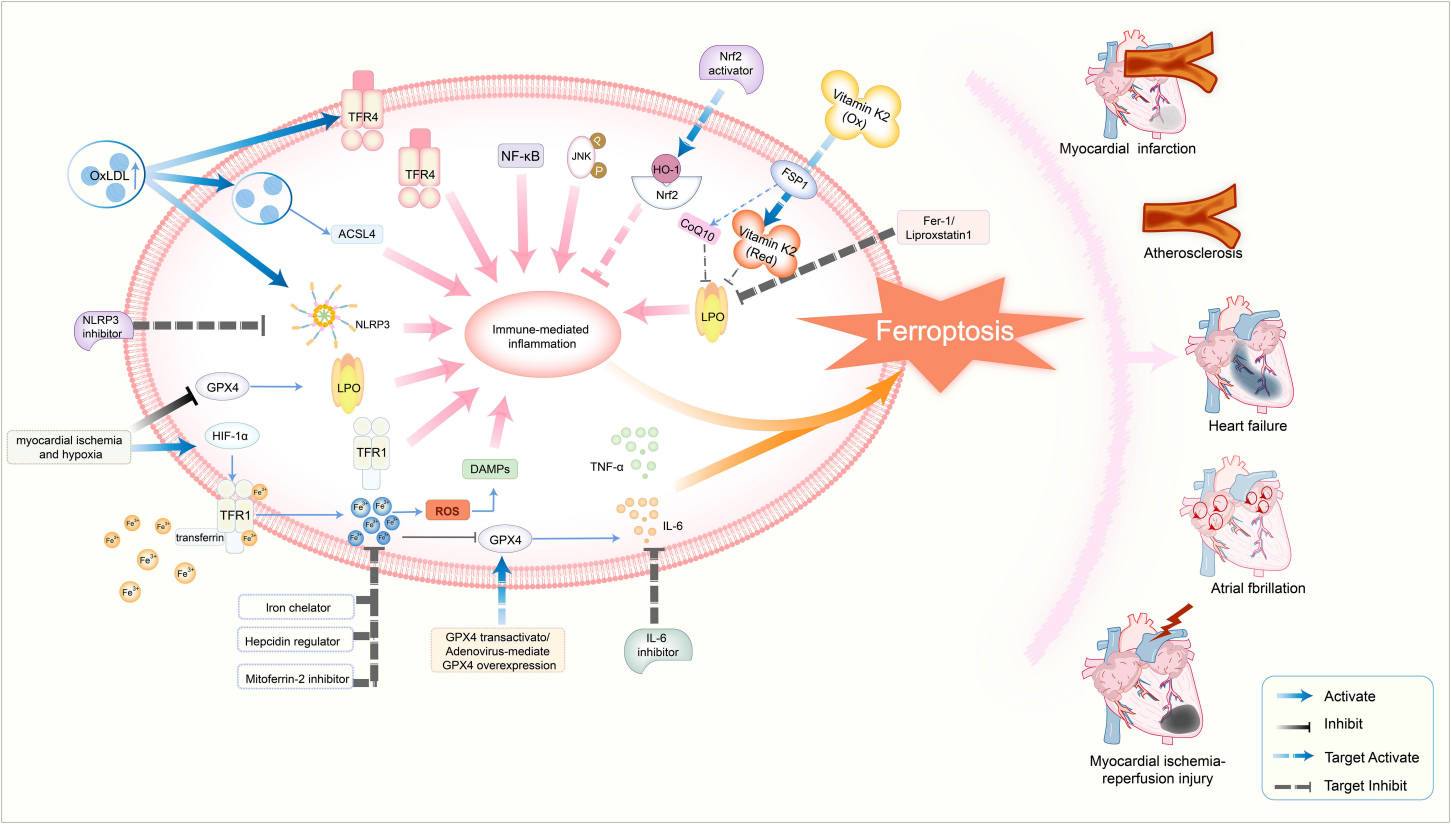
Schematic diagram illustrating the core mechanisms of ferroptosis–immune interactions in cardiovascular diseases and their therapeutic targets. This figure summarizes the vicious cycle in which ferroptosis and immune-mediated inflammation mutually reinforce each other, collectively driving the progression of various cardiovascular diseases initiated by stimuli such as myocardial ischemia and hypoxia. Solid arrows represent the principal molecular mechanisms and signaling pathways involved in disease pathogenesis, including ferroptosis (e.g., TFR1, GPX4, and LPO), immune-mediated inflammation (e.g., DAMPs, TNF-α, and NF-κB pathway), and their intersections. Dashed arrows denote therapeutic interventions targeting critical pathways, such as NLRP3 inhibitors, GPX4 transactivators, and ferroptosis inhibitors. Disease outcomes: This pathogenic network culminates in cardiovascular disorders, including myocardial infarction, myocardial ischemia–reperfusion injury, heart failure, and atrial fibrillation. This figure was created using BioRender (biorender.com).

In summary, the interplay between ferroptosis and inflammation synergistically drives aortic medial degeneration, playing a critical role in the pathological progression of aortic aneurysms and dissections. **Figure 5** illustrates the core mechanisms of ferroptosis–immune interactions in cardiovascular diseases and their associated therapeutic targets.

## 5 Therapeutic strategies and prospects for targeting the ferroptosis and immune–inflammation in CVDs

### 5.1 Iron metabolism modulators

Deferoxamine is a classic iron chelator that is widely used to reduce tissue iron accumulation and deposition. It has been shown to decrease plaque burden in AS models (106). Combined treatment with N-acetylcysteine and deferoxamine improves cardiac function in Wistar rats following non-reperfusion acute myocardial infarction (AMI) (142). Deferoxamine protects against MIRI by chelating iron and inhibiting ferroptosis (143). Yang et al. reported that deferoxamine combined with sevoflurane post-treatment attenuated MIRI by restoring mitochondrial autophagy via HIF-1/BNIP3 signaling in GK rats (144). A systematic evaluation by Lamichhane indicated that deferoxamine reduces myocardial oxidative stress by inhibiting iron-dependent LPO and ROS generation, protecting cardiomyocytes during extracorporeal circulation procedures (CPB) (145). These studies strongly support the potential clinical application of deferoxamine.

Deferiprone, an orally active bidentate hydroxypyridone iron chelator, forms stable iron complex. It exhibits anti-inflammatory properties and reduces fibroblast migration *in vitro* (146). Long-term deferiprone treatment improves myocardial magnetic resonance imaging findings and reduces the risk of developing or worsening heart disease (147). Sarina Entezari et al. reviewed iron chelation therapy, noting that deferiprone effectively penetrates cell membranes, removes excess cardiac iron, offers better cardioprotection than deferoxamine, and improves left ventricular ejection fraction (148).

Ferrostatin-1 (Fer-1) is a potent and selective ferroptosis inhibitor that prevents cell death by blocking lipid membrane damage through a reductive mechanism. Fer-1 partially inhibits iron deposition and LPO, alleviating atherosclerotic lesions in ApoE^-^/^-^ mice fed a high-fat diet (107). It suppresses cardiomyocyte ferroptosis post-MI through Nrf2 pathway activation (149) and significantly reduces MIRI in rats by lowering Fe²^+^ levels, decreasing ACSL4 expression, and attenuating mitochondrial damage (150). In a hypoxia/reoxygenation model simulating MIRI, treatment with Fer-1 significantly improved the viability of neonatal mouse cardiomyocytes 24 h after reoxygenation (151). In SIRT3 conditional knockout (cKO) mice, Fer-1 targeted mitochondrial iron–sulfur clusters and ouabain-sensitive enzymes, improving cardiac function and ameliorating HF over 14 days (152). Moreover, Fer-1-mediated inhibition of ferroptosis improves AF outcomes (153).

Liproxstatin-1 is a selective ferroptosis inhibitor. Su et al. observed that male ApoE^-^/^-^ mice with radiation-associated atherosclerosis (RAA) exhibited increased plaque burden, endothelial cell loss, and elevated LPO, all of which were ameliorated by Liproxstatin-1, suggesting its potential in AS management (154). Chengshen et al. demonstrated that Liproxstatin-1 modulates post-MI ferroptosis and immune responses, reducing macrophage (CD68) counts, promoting M2 polarization, diminishing inflammation, decreasing myocardial fibrosis and markers such as α-SMA, collagen I, and collagen III, ultimately improving cardiac remodeling in rats. Collectively, these findings indicate that Liproxstatin-1 represents a potential therapeutic agent for mitigating adverse remodeling following MI (155). Additionally, Liproxstatin-1 protects against MIRI by restoring GPX4 levels and reducing voltage-dependent anion channel 1 (VDAC1) expression (156).

Mitochondrial iron transport protein (mitoferrin-2), a mitochondrial iron transport protein, is an emerging therapeutic target. Inhibition of this pathway attenuates iron-induced ferroptosis and STING-dependent inflammation, as demonstrated in preclinical studies (157). Furthermore, Wang et al. reported that mitoferrin-2 deficiency prevents mitochondrial endothelial injury caused by iron overload and attenuates AS (158).

In summary, although iron chelators show therapeutic potential in CVDs, their clinical translation remains challenging. Key obstacles include limited translatability from animal models to humans and a lack of long-term safety data beyond short-term efficacy assessments.

Hepcidin, a liver-secreted peptide hormone, is a central regulator of systemic iron homeostasis. It binds to the iron efflux protein ferroportin and inhibits cellular iron export. Elevated serum iron levels stimulate hepcidin secretion, reducing iron release from cells into the serum and extracellular fluid. Conversely, low hepcidin levels increase iron export from hepatocytes and macrophages, increasing plasma iron and transferrin saturation (159). Hepcidin modulation affects iron metabolism and has direct implications for CVD. In patients with stable coronary artery disease, hepcidin levels are negatively correlated with total and cardiovascular mortality, suggesting that lower hepcidin levels may increase CVD risk (160). Reducing hepcidin may enhance iron absorption and utilization, alleviating anemia and potentially benefiting patients with CVD (161). Aboelsaad et al. reported that lower iron-regulatory hormone levels were linked to a higher risk of HF, particularly HF with preserved ejection fraction (HFpEF), and diastolic dysfunction in older adults without anemia (162). However, the precise role of ferritin in AS pathogenesis remains unclear (163).

### 5.2 Antioxidant defense enhancers

#### 5.2.1 GPX4-targeted interventions

GPX4-targeted interventions include GPX4 transactivators and adenovirus-mediated GPX4 overexpression. GPX4 transactivators enhance GPX4 activity by binding to its transactivation site. By increasing GPX4 activity, these agents efficiently scavenge LPOs, inhibit the initiation and progression of ferroptosis, and promote cell survival, particularly in cells that are susceptible to oxidative stress. A GPX4 variant activator mitigated myocardial injury in a mouse model of adriamycin-induced cardiotoxicity. Consequently, GPX4 activation represents a promising therapeutic approach for treating myocardial injury (164).

Adenovirus-mediated GPX4 overexpression has been reported to significantly attenuate STING-induced ferroptosis and improve cardiac functional recovery in MIRI (165). Additionally, GPX4 targeting alleviated ferroptosis and delayed AAA formation. GPX4 also modulates macrophage/monocyte migration and activation in AAA in mouse tissues. *In vitro* studies have further demonstrated that GPX4 overexpression inhibits IL-6-induced activation of the JAK1–STAT3 signaling pathway in VSMCs, thereby reducing the generation of pro-inflammatory macrophages (166).

Collectively, experimental evidence demonstrates that both GPX allosteric activators and GPX4 overexpression effectively suppress ferroptosis and inflammation, underscoring their therapeutic potential in CVDs. However, the potential toxicity associated with sustained GPX4 overexpression requires careful consideration of the therapeutic strategy. Moreover, although studies using adenoviral vectors have shown efficacy in animal models, their translatability to humans and the risk of provoking robust immune responses warrant further investigations.

#### 5.2.2 FSP1–CoQ10 system

Ferroptosis-suppressor protein 1 (FSP1) is a recently identified ferroptosis inhibitor that operates independently of GPX4. FSP1 enhances cellular resistance to ferroptosis via the coenzyme Q10 (CoQ10) system. CoQ10, a lipid-soluble antioxidant, supports FSP1 activity by catalyzing the reduction of ubiquinone (CoQ10) to ubiquinol (CoQ10-H2) and trapping free radicals generated during LPO. This NAD(P)H-dependent reaction in the cellular membrane is critical for ferroptosis resistance (167). Its activity depends on N-terminal myristoylation, which facilitates membrane localization and more effective ferroptosis inhibition (168, 169).

Vitamin K2, a cofactor of FSP1, is a potent ferroptosis inhibitor. It may confer protection against AS and vascular calcification through multiple mechanisms (170), including the inhibition of VSMC calcification and anti-apoptotic effects via the GAS6–AXL pathway (171). The CoQ10 analog idebenone reduces cardiac hypertrophy in Friedreich’s ataxia patients and demonstrates cardioprotective effects (172). Pan et al. reported that CoQ10 attenuates macrophage-mediated cardiac inflammation following MI through the NLRP3/IL-1β pathway. Plasma CoQ10 levels were lower in MI patients than in controls (0.46 ± 0.10 vs. 0.76 ± 0.31 μg/mL). CoQ10 supplementation significantly enhanced cardiac functional recovery at 1- and 3-months post-PCI. In mouse models, MI mice treated with CoQ10 showed improved survival (61.90% vs. 42.85%) and marked attenuation of myocardial fibrosis, hypertrophy, and dysfunction compared with vehicle-treated controls. CoQ10 also significantly reduced the recruitment of pro-inflammatory CCR2+ macrophages to the infarcted myocardium and suppressed NLRP3–IL-1β pathway activation. This suggests that CoQ10 promotes early post-MI cardiac recovery by modulating macrophage-mediated inflammation (173). CoQ10 has also been extensively studied in HF, where it improves mitochondrial function and antioxidant defense and mitigates oxidative stress (174).

#### 5.2.3 Nrf2 activator

The Nrf2 activator bardoxolone methyl protects against MIRI by reducing oxidative stress and cardiomyocyte apoptosis via the activation of the Nrf2–HO-1 signaling pathway (175). Similarly, bardoxolone attenuates oxidative stress and inflammatory responses through Nrf2 activation, providing protective effects in chronic HF patients (176). McCullough reviewed bardoxolone’s beneficial effects on cardiovascular and renal functions in patients with type 2 diabetes mellitus and chronic kidney disease (177). Collectively, these studies suggest that bardoxolone may serve as a therapeutic agent for CVD, particularly through its capacity to mitigate oxidative stress and inflammatory responses.

### 5.3 Inflammation-targeted interventions

#### 5.3.1 NLRP3 inhibitors

MCC950 is a potent and selective NLRP3 inhibitor. Zeng et al. reported that MCC950 reduces the serum levels of pro-inflammatory cytokines, including TNF-α, IL-1β, IL-6, and IL-18, by suppressing NLRP3 inflammasome activation. This attenuation of inflammatory responses and pyroptosis in AS models subsequently mitigates disease progression (178). MCC950 also decreases AS plaque size and macrophage content without affecting the serum lipid levels (178). Zheng et al. found that MCC950 alleviated post-resuscitation myocardial dysfunction and improved survival in rat models of cardiac arrest and resuscitation (179). In a mouse model of MI, MCC950 significantly reduced myocardial fibrosis, improved cardiac remodeling, and enhanced cardiac function, highlighting its potential therapeutic effect during post-MI recovery (180). Furthermore, MCC950 mitigated isoproterenol-induced cardiac dysfunction by inhibiting cardiomyocyte senescence and oxidative stress. MCC950 restored ejection fraction, reduced cardiac hypertrophy, and improved cardiac function in ISO-treated mice (181). Collectively, these studies demonstrate that MCC950, a selective NLRP3 inflammasome inhibitor, holds significant therapeutic potential in AS, MI, and HF models.

Dapansutrile is an oral small-molecule selective NLRP3 inhibitor with a favorable safety and tolerability profile in HF patients, though its efficacy requires further evaluation. A Phase 1B randomized, double-blind, dose-escalation, single-center study assessed the safety and pharmacodynamics of patients with stable HFrEF (New York Heart Association Classes II–III). Subjects were randomized in a 4:1 ratio to receive dapansutrile for up to 14 days across three sequential ascending dose cohorts (500 mg, 1,000 mg, or 2,000 mg), with 10 patients per group. Clinical assessments, biomarker measurements, transthoracic echocardiography, and maximal cardiopulmonary exercise testing were performed at baseline, day 14, and day 28. Thirty participants (20 males) completed 13 (12–14) days of treatment. The results revealed that, compared with baseline, the clinical and laboratory parameters on day 14 indicated stability, with no significant differences within the combined dapansutrile group or across the dose cohorts. In the dapansutrile cohort, left ventricular ejection fraction improved from 31.5% (27.5–39.0%) to 36.5% (27.5–45, P = 0.039), and exercise time increased from 570 s (399.5–627.0 s) to 616 s (446.5–688.0 s, P = 0.039) (2). Thus, these findings provide preliminary safety evidence for NLRP3 inhibition in HFrEF patients who may be intolerant to nonsteroidal anti-inflammatory drugs (NSAIDs) or glucocorticoids. Additionally, Aliaga et al. demonstrated that dapansutrile preserves adrenergic responsiveness and prevents left ventricular diastolic dysfunction in a mouse model of reperfused anterior wall MI, suggesting its potential to prevent HF development in ischemic cardiomyopathy (182). Yang et al. reported that dapansutrile treatment ameliorated HFpEF and reduced atrial fibrosis and inflammation in a rat model of HF (183). These findings suggest that dapansutrile represents a promising yet unproven therapeutic approach for HF, and its efficacy and clinical value require further validation in multicenter, large-scale trials.

#### 5.3.2 IL-6 inhibitor

Tocilizumab, an anti-IL-6 receptor antibody, shows potential for treating CVDs. It may improve cardiac function by inhibiting inflammatory responses and attenuating myocardial injury following MI (184), and may enhance myocardial salvage in patients with acute ST-segment elevation myocardial infarction (STEMI) (185).

Pottebaum et al. investigated tocilizumab in antibody-mediated rejection (AMR) after heart transplantation. Tocilizumab markedly improved biventricular systolic function, likely by reducing endothelial and intravascular macrophage activation and cytokine production, providing preliminary evidence of its therapeutic potential in HF (186). In the randomized, double-blind, placebo-controlled ASSAIL-MI trial, tocilizumab significantly increased the Myocardial Salvage Index (MSI) in STEMI patients, while also elevating IL-6, IL-8, and IL-1ra levels. These findings suggest that tocilizumab may confer cardioprotection by mitigating ischemia–reperfusion injury in STEMI (187) (**Table 1**).

**Table 1:** Drugs and small molecule inhibitors targeting the ferroptosis-immune-mediated inflammation axis in the treatment of CVDs.

Drugs or small molecule inhibition	Disease	Mechanism	Model/Patients	Key findings	Clinical trial status	References
Deferoxamine	MIRI	Restored HIF-1/BNIP3-mediated mitochondrial autophagy	GK rat MIRI model/SD rat MIRI model	DFO treatment combined with SPostC could alleviate MIRI in diabetic rats.	/	(144)
	MIRI	Inhibits iron-dependent lipid peroxidation and reactive oxygen species (ROS) production	patients	1.DFO decreases in ROS production. 2.Protects cardiac tissue from oxidative damage during CPB. 3. Improve left ventricular ejection fraction and wall motion score index.	/	(145)
Ferrostatin-1	AS	1.Partially inhibited the levels of SLC7A11 and GPX4 2.Down-regulated the expressions of adhesion molecules and up-regulated eNOS expression	ApoE^−/−^ mouse AS model/MAECs.	1. Fer-1 alleviated AS lesion in HFD-fed ApoE^−/−^ mouse. 2.Increased cell viability and reduced cell death in ox-LDL-treated MAECs.	/	(107)
	AMI	1.Inhibited cardiomyocyte ferroptosis after MI by activating Nrf2 signaling 2.Improvedferroptosis and changes in Nrf2, xCT, and GPX4 expressions 3.Nrf2 deficiency increased myocardial injury after MI	C57 mouse MI model, Nrf2^−/−^ mouse MI model, /H9C2 ferroptosis cell model	1. Fer-1 improves cardiac function and protects against myocardial injury caused by MI. 2.Nrf2 signaling plays a crucial part in regulating cardiomyocyte ferroptosis after MI.	/	(149)
	MIRI	Reduced Fe2+ concentrations and the production of peroxides	SD rat MIRI model	Fer-1 significantly reduced myocardial I/R injury, Fe2+ concentrations and ACSL4 expression and attenuated mitochondrial impairment.	/	(150)
	I/R	Inhibited ferroptosis	C57BL6J mouse MIRI model/NMCM H/R model	Fer-1 improved myocardial cell viability after 24 h of reoxygenation.	/	(151)
	HF	1.Upregulates expression of mitochondrial GPX4 and aconitase in SIRT3cKO mouse 2.Reverses SIRT3cKO-induced decreases in mitochondrial aconitase activity and cardiac calcium dysregulation	SIRT3cKO mouse/SIRT3loxp mouse HF model	Fer-1 inhibited ferroptosis and ameliorated cardiac dysfunction by specifically targeting mitochondrial aconitase and iron-sulfur clusters.	/	(152)
	AF	Targets mitochondrial iron-sulfur clusters and aconitase to improve cardiac function in Sirtuin 3 cardiomyocyte knockout mouse	C57BL/6J mouse AF model	Significant improvement for AF.	/	(153)
Liproxstatin-1	RAA	1.Alleviated inflammation cytokines after Irradiation 2.Inhibited ferroptosis through the Nrf2/GPX4 pathway	ApoE^−/−^ mouse RAA model/HAECs	1.Liproxstatin-1 alleviated plaque burdens of RAA mouse. 2. Ameliorated radiation-induced endothelial injury and lipid peroxidation in RAA.	/	(154)
	MI	Decreased fibrosis and reduces levels of α-SMA, Collagen I, and Collagen III proteins	SD rat MI model	1.Liproxstatin-1 reduced macrophage (CD68+) counts, promotes M2 polarization decreased inflammation. 2. Improved cardiac function.	/	(155)
	MIRI	Reduced VDAC1 levels and restores GPX4 levels	C57BL/6J mouse MIRI model	1.Liproxstatin-1 reduced myocardial infarct size and protected mitochondrial structural integrity. 2.Liproxstain-1 protected the mouse MIR.	/	(156)
Mitoferrin-2	AS	1.Mfrn2 silencing reversed TNF-α-induced mitochondrial iron overload and ROS production in MAECs 2. Mfrn2 silencing relieved TNF-α-induced mitochondrial dysfunction and fission	ApoE^−/−^ mouse AS model	1.Mfrn2 deficiency attenuates endothelial dysfunction by decreasing iron levels within the mitochondria and mitochondrial dysfunction. 2. Mfrn2 deficiency prevents mitochondrial iron overload-induced endothelial injury and alleviates atherosclerosis.	/	(158)
Hepcidin	HF	no	CAD patients and no-CAD patients	1.In stable patients undergoing angiography, serum iron, transferrin saturation, sTfR, and ferritin had J-shaped associations and hemoglobin only a marginal association with cardiovascular and total mortality. 2. Hepcidin was continuously and inversely related to mortality.	/	(160)
	CKD	no	CKD patients	Among early CKD patients with FID, there was high level of hepcidin along with other inflammatory parameters, which may be associated with poor cardiovascular disease outcome due to increased inflammation.	/	(161)
	HF	no	Adults from the ongoing, longitudinal Atherosclerosis Risk in Communities	In community-dwelling older adults without anemia, lower plasma hepcidin levels associate with a higher risk of incident HF (particularly HFpEF) and diastolic dysfunction.	/	(162)
GPX4 allosteric activator	Doxorubicin-induced myocardial injury	Inhibited ferroptosis by activating GPX4	Doxorubicin-induced myocardial injury mouse model/HT1080 cells	GPX4 allosteric activators inhibit ferroptosis and exert myocardial protection in doxorubicin-induced myocardial injury mouse model.	/	(164)
Vitamin K2	Vascular calcification	Through restoring the Gas6/AxL/Akt anti-apoptotic pathway	VSMC from SD rat	VK2 significantly inhibited CaCl2- and β-sodium glycerophosphate (β-GP)-induced VSMC calcification and apoptosis.	/	(171)
Idebenone	Friedreich’s ataxia	no	Friedreich’s ataxia patients	Idebenone is effective at controlling cardiac hypertrophy in Friedreich’s ataxia.	/	(172)
Coenzyme Q10	MI	Partially through inhibiting NLRP3/IL1β pathway-mediated inflammation in macrophages	MI patients/C57BL/6 mouse MI model /Thioglycollate-elicited macrophages were isolated from the peritoneal cavity of mouse	1.CoQ10 supplementation promotes early recovery of cardiac function in MI patients post PCI. 2. Single CoQ10 treatment alleviates adverse cardiac remodeling in response to experimental MI. 3. CoQ10-treated macrophages exhibit a less inflammatory state and suppressed activation of the NLRP3/IL1β pathway. 4. CoQ10 mitigates post-infarction cardiac inflammation associated with IL1β signaling.	Phase II clinical trial	(173)
Bardoxolone methyl	MIRI	Inhibited oxidative stress and cardiomyocyte apoptosis via activating the Nrf2/HO-1 pathway	SD rat MIRI model	Bardoxolone methyl ameliorated myocardial I/R injury.	/	(175)
	MI	1.Mediated Nrf2 activation and upregulated of antioxidant enzymes 2.Inhibits the production of inflammatory cytokines	SD rat CHF model	Systemic activation of Nrf2 and antioxidant protein signaling by bardoxolone methyl may have beneficial effects on cardiac function and result in improvements by enhancing antioxidant enzyme expression and attenuating myocardial inflammation.	/	(176)
MCC950	AS	Inhibited the activation of NLRP3/ASC/Caspase-1/GSDMD-N axis.	ApoE^−/−^ mouse/myeloid leukemia mononuclear cells/THP-1 cells	1.MCC950 attenuated atherosclerotic plaque formation. 2. mitigated NLRP3 mediated macrophages pyroptosis within AS plaque.	/	(178)
	CA	1.Reduces circulatory IL-1β and cTnI plasma level 2. decreases expression levels of the principal constituents of NLRP3 inflammasome within the left ventricle	SD rat CA and CRP model	MCC950 mitigates post-resuscitation myocardial dysfunction and improves survival in a rat model of cardiac arrest and resuscitation.	/	(179)
Dapansutrile	HF	no	HF patients	Treatment with dapansutrile for 14 days was safe and well tolerated in patients with stable HFrEF.	Phase IB clinical trial	(2)
	Severe ischemic cardiomyopathy	Inhibits aberrant NLRP3 pro-inflammatory signaling	ICR mouse non-reperfused AMI model	Dapansutrile could preserve contractile reserve and diastolic function in a mouse model of severe ischemic cardiomyopathy due to non-reperfused anterior wall myocardial Infarction.	/	(182)
	AF	Inhibited activation of the NLRP3 inflammasome	DSS rat HF model	Dapansutrile ameliorates atrial inflammation and vulnerability to atrial fibrillation in HFpEF rats.	/	(183)
Tocilizumab	STEMI	no	Patients admitted with STEMI within 6 h of symptom onset	Tocilizumab increased myocardial salvage in patients with acute STEMI.	Phase II clinical trial	(185)
	NSTEMI	no	NSTEMI patients who were scheduled for coronary angiography	Tocilizumab increases citrullinated histone 3 in non-ST segment elevation myocardial infarction.	Phase II clinical trial	(184)
	Acute cardiac Antibody-mediated rejection (AMR)	no	Patients with acute cardiac AMR	Tocilizumab significantly improved both ventricular systolic function after 4 to 6 months of treatment.	/	(186)
	STEMI	no	STEMI patients	1.Tocilizumab increased IL-6, IL-8 and IL-1ra in patients with STEMI. 2. Attenuates the correlation of IL-6 and IL-8 with neutrophils/C reactive protein.	Phase II clinical trial	(187)

SPostC, sevoflurane postconditioning; CPB, cardiopulmonary bypass; DFP, Deferiprone; Fer-1, Ferrostatin-1; AS, Atherosclerosis; GPX4, glutathione peroxidase 4; AMI, acute myocardial infarction; AMI, acute myocardial infarction; MI, myocardial infarction; MIRI, Myocardial Ischemia/Reperfusion Injury; I/R, ischemia/reperfusion injury; HF, heart failure; AF, atrial fibrillation; RAA, radiation-associated atherosclerosis; Mfrn2, mitoferrin-2; CAD, coronary artery disease; CKD, chronic kidney disease; HFpEF, heart failure with preserved ejection fraction; AAA, abdominal aortic aneurysm; VSMC, vascular smooth muscle cell; OP, Osteoporosis; CHF, chronic heart failure; eNOS, endothelial nitric oxide synthase; CBP, CREB-binding protein; CRP, cardiopulmonary resuscitation; HFrEF, heart failure with reduced ejection fraction; CRP, C-reactive protein; STEMI, ST-segment elevation myocardial infarction; NSTEMI, non-ST segment elevation myocardial infarction; ASTEMI, acute ST-segment elevation myocardial infarction; AMR, antibody-mediated rejection; GK, Goto-Kakizaki; MAECs, mouse aortic endothelial cells; NMCM, neonatal mouse cardiomyocytes; FID, functional iron deficiency; HT1080 cells, human fibroblasts and sarcoma cells; VC, vascular calcification; CHF, chronic heart failure; CA, cardiac arrest; DSS, Dahl salt-sensitive; ICR, Institute of Cancer Research; HAECs: Human artery endothelial cell line; AMR, antibody-mediated rejection; HAECs:Human artery endothelial cell line.

In summary, whether through iron chelators, enhancers of antioxidant defenses, or inhibitors of inflammasomes and immune-inflammatory mediators, these interventions hold potential protective effects against CVDs. However, limitations such as reliance on animal models, small sample sizes in human studies, and incomplete understanding of specific mechanisms leave some findings unresolved or contradictory to each other. Therefore, more extensive studies are warranted.

### 5.4 Traditional Chinese medicine and its monomers

Traditional Chinese medicine (TCM) has played a pivotal role in Chinese healthcare for thousands of years. Several herbs and bioactive compounds, including ginseng, *Pueraria mirifica*, curcumin, astragaloside IV, and resveratrol, have been shown to inhibit ferroptosis and modulate immune-mediated inflammation in CVD.

#### 5.4.1 Ginseng

Ginseng, cultivated predominantly in China’s Changbai Mountain region, exhibits potent antioxidant and anti-inflammatory properties, enhances immune function, and acts as a ferroptosis inhibitor in the body. Ginsenoside Re mitigates MIRI and suppresses ferroptosis via the miR-144-3p–SLC7A11 axis (188). Ginsenoside Rg3 attenuates cardiac MIRI-induced ferroptosis via the KEAP1–Nrf2–GPX4 signaling pathway (189). Ginsenoside Rb1 protects against adriamycin-induced cardiotoxicity by inhibiting autophagy and ferroptosis (190). Additionally, Ginsenoside Rg1 inhibits macrophage polarization, thereby reducing MIRI associated with cardiac inflammation (191). *Panax quinquefolius* L. saponins suppress the TLR4–MyD88–NF-κB pathway, inhibiting NLRP3 inflammasome activation and protecting against MIRI from no-flow injury (192). Pretreatment with ginsenoside Rg3 also inhibited the RhoA–ROCK pathway, further attenuating ferroptosis and cardiac injury induced by high-altitude, low-pressure hypoxia in mice (193). Moreover, ginseng exerts cardioprotective effects post-MI by reducing myocardial fibrosis and inflammation via the SIRT1 signaling pathway (194). In summary, the experiments indicate that ginseng and its monomers exert a protective effect against cardiovascular system diseases by inhibiting ferroptosis and reducing inflammation.

#### 5.4.2 Puerarin

Puerarin, a flavonoid glycoside extracted from the root of wild kudzu (*Pueraria lobata*), promotes blood circulation, alleviates blood stasis, enhances microcirculation, dilates coronary and cerebral vessels, and reduces myocardial oxygen consumption. In aortic tissue and serum from AS mice, puerarin downregulated α-SMA and inflammatory cytokines IL-6 and IL-8, suppressing VSMC proliferation and inflammation, potentially through the miR-29b-3p–IGF1 pathway (195). Puerarin-V protects the myocardium from isoprenaline-induced MI in mice via modulation of the PPAR-Υ–NF-κB pathway (196). Puerarin downregulates VDAC1, inhibiting ferroptosis and protecting against MIRI (197). Additionally, it suppresses NLRP3 inflammasome activation to prevent MIRI, likely via the SIRT1–NF-κB pathway (198). Puerarin mitigates pressure overload-induced HF by alleviating ferroptosis (125) and protects against sepsis-induced MI through AMPK-mediated ferroptosis signaling (199). Collectively, these findings identify puerarin as a protective agent against AS and MIRI by modulating inflammatory and ferroptotic pathways, thereby preventing subsequent HF.

#### 5.4.3 Curcumin

Curcumin, a natural phenolic antioxidant derived from turmeric rhizomes (*Curcuma longa*), exhibits anti-inflammatory effects relevant to AS therapy (200). It restores cholesterol transport homeostasis and modulates inflammatory responses in M1 macrophages, preventing AS progression (201). Curcumin may also regulate the balance between M1 and M2 macrophages during AS treatment (202). In a rat MI model, curcumin mitigated cardiotoxicity and improved myocardial function (203). Pretreatment with curcumin enhances cardiac contractility and attenuates myocardial and renal injuries by reducing inflammation and oxidative stress (204). Curcumin also alleviates MIRI by inhibiting ferroptosis, autophagy, and apoptosis via the HES1 pathway (205) and SIRT1–AKT–FoxO3a axis (8). Ceria nano-enzymes conjugated with curcumin can attenuate sepsis-induced cardiac injury by suppressing ferroptosis and inflammation (206). Furthermore, curcumin reduces the development of thoracic aortic aneurysms by inhibiting inflammation and vascular endothelial growth factor (VEGF) expression (207). These findings indicate that curcumin exerts protective effects against CVDs by inhibiting ferroptosis and modulating anti-inflammatory pathways.

#### 5.4.4 Astragaloside IV

Astragalus (*Astragalus membranaceus*) enhances spleen and middle Jiao function, elevates Yang, and supports immune defense. Astragaloside IV, the principal bioactive component, exhibits anti-inflammatory, cardioprotective, and antifibrotic effects. It ameliorates myocardial fibrosis, suppresses inflammatory responses, reduces oxidative stress, regulates myocardial energy metabolism, improves contractility, and prevents cardiomyocyte apoptosis (208–211). Astragaloside IV modulates the MAPK–NF-κB signaling pathway to alleviate AS in LDL receptor-deficient (Ldlr^−/−^) mice (212), and inhibits the TLR4–MyD88–NF-κB pathway to prevent acute MI (213). Combined treatment with astragaloside IV and tanshinone IIA inhibits the STING pathway, reducing MIRI (214), and improving myocardial metabolism, and decreasing inflammation in HFpEF mice (215). Astragaloside IV also attenuates CD36-mediated ferroptosis, improving myocardial function in DCM rats (216) and mitigating 3,4-benzopyrene-induced AAAs by modulating macrophage-driven inflammation (217). In summary, Astragaloside IV preserves cardiovascular function by exerting anti-inflammatory effects and inhibiting ferroptosis.

#### 5.4.5 Resveratrol

Resveratrol, a stilbene polyphenol, exerts cardioprotective, antioxidant, anti-inflammatory, antiviral, hepatoprotective, antitumor, and immunomodulatory effects. Pitavastatin–resveratrol bio-nanocomplexes counteract hyperhomocysteinemia-induced AS by blocking ferroptosis-associated lipid deposition (218). Resveratrol regulates the KAT5–GPX4 pathway to inhibit ferroptosis, thereby reducing cardiac injury in a rat MI model (219). It mitigates oxidative stress and suppresses ferroptosis, thereby providing protection against MIRI (220). Similarly, modulation of the VDAC1–GPX4 pathway alleviates ferroptosis and protects against MIRI (221). Resveratrol also inhibits ferroptosis and slows HF progression via SIRT1/p53 activation (222). However, direct evidence that resveratrol improves atrial myocardial fibrosis or prevents atrial fibrillation remains insufficient (131). Regarding its anti-inflammatory effects, long-term resveratrol administration reduces monocyte infiltration and attenuates angiotensin II (AngII)-induced atherosclerotic plaque formation in ApoE^−/−^ mice (223). Additionally, resveratrol suppresses inflammation via the TLR4–NF-κB pathway, mitigating MIRI in rats (224), and improves cardiac function in patients with systolic HF by modulating inflammatory processes (225). Furthermore, the upregulation of HMOX1 by resveratrol decreases extracellular matrix degradation, apoptosis, autophagy, and inflammation in vascular smooth muscle cells, thereby inhibiting the progression of AAA (226) (**Table 2**).

**Table 2:** Traditional Chinese medicine and its monomer components targeting the ferroptosis-immune-mediated inflammation axis in the treatment of CVDs.

Traditional Chinese medicine and its monomer components	Disease	Mechanism	Model/Patients	Key findings	References
Ginsenoside Re	MIRI	Regulated miR-144-3p/SLC7A11 level	WKY rat MIRI model/H9c2 cells/endothelial progenitor cells (EPCs)	1. Ginsenoside Re significantly reduced cardiac damage caused by ferroptosis during MI/RI and glutathione decline.	(188)
Ginsenoside Rg3	MIRI	1. Activated the Nrf2 signaling pathway 2. Regulated the keap1/Nrf2 signaling pathway	C57BL/6 mouse MIRI model/OGD/R H9c2 cell model	1. Ginsenoside Rg3 improved cardiac function and infarct size in mouse with MI/RI. 2. Increased the expression of the ferroptosis-related protein GPX4 and inhibited iron deposition in mouse with MI/R injury. 3. Attenuate OGD/R-induced ferroptosis in H9C2 cells.	(189)
Ginsenoside Rg1	MIRI	Inhibited macrophage M1 polarization by inhibiting AIM2 inflammasome activation	SD rat MIRI model/BMDM	1. Rg1 significantly attenuated myocardial inflammation, inhibited M1 macrophage polarization and reduced cardiac fibrosis during MI/R injury. 2. Inhibited the activation of the AIM2 inflammasome *in vitro*.	(191)
*Panax quinquefolius* L. Saponins (PQS)	MIR No-Reflow (NR)	Inhibited the activation of NLRP3 inflammasome via TLR4/MyD88/NF-κB signaling	Wistar rat MIRI model	1. PQS reducing ischemia, infarction and NR area; 2. improving cardiac function; 3. preventing pathological morphology changes of myocardium; 4. depressing leukocytes’ aggregation and adhesion.	(192)
Ginsenoside Rg3	Cardiac injury induced by high-altitude hypobaric hypoxia exposure	Suppressed ferroptosis through inhibition of the RhoA/ROCK signaling pathway	HACI C57 BL/6J mouse model	1. Ginsenoside Rg3 mitigates cardiac injury induced by high-altitude hypobaric hypoxia exposure. 2. Reduces inflammation and ferroptosis	(193)
Panax ginseng	MI	1. Promoted SIRT1 expression 2. Significantly suppressed NLRP3-caspase1 inflammasome and TGFBR1/Smads signaling	C57BL/6 mouse MI model/CFs and RAW264.7 cells	*Panax ginseng* attenuated myocardial fibrosis and inflammation and protect cardiac function after myocardial infarction.	(194)
Ginsenoside Rb1	Doxorubicin induced cardiotoxicity	Suppressed autophagy and ferroptosis	DIC C57BL/6 mouse model	Rb1 attenuated Dox induced cardiac dysfunction, myocardium hypertrophy and interstitial fibrosis.	(190)
puerarin	AS	Regulated miR-29b-3p/IGF1 pathway	APoE^−/−^ mouse AS model/hVSMC AS cell model	1. Puerarin reduced the expression of α-SMA and the inflammatory proteins. 2. Decreased hVSMC proliferation, and migration.	(195)
Puerarin-V	MI	Regulated PPAR-Υ/NF-κB Pathway. activated of PPAR-γ, inhibited NF-κB signaling	Isoproterenol (ISO)-induced C57BL/6 mouse MI model/human coronary artery endothelial cell (HCAECs)	1. Puerarin-V exerts cardioprotective effects against ISO-induced MI in mouse. Puerarin-V significantly improves ventricular wall infarction, decreases the incidence of mortality, and inhibits the levels of myocardial injury markers. 2. Reduced cell death and suppressed the inflammation cytokines expression.	(196)
Puerarin	MIRI	Inhibited ferroptosis through downregulation of VDAC1	C57BL/6 mouse MIRI model/H9c2 cell H/R model	Pretreatment with puerarin mitigated cardiomyocyte ferroptosis, restored redox balance, preserved mitochondrial energy production and maintained mitochondrial function following MI/RI.	(197)
	MIRI	1. Inhibited the NLRP3 inflammasome 2. Regulated the SIRT1/NF-κB pathway	C57BL/6 mouse MIRI model	1. Puerarin reduced myocardial infarct size, serum CK-MB activity, and apoptotic cell death, and improved cardiac structural damage and dysfunction. Puerarin protected against MI/R injury.	(198)
	HF	1. Regulation of Nox4 signaling 2. Inhibited ferroptosis	SD rat HF model/H9c2 cells	Puerarin inhibited myocyte loss during HF.	(125)
	Sepsis-induced myocardial injury	Regulated AMPK-mediated ferroptosis signaling	SD rat sepsis-induced myocardial injury model	Puerarin protects against sepsis-induced myocardial injury.	(199)
Curcumin	AS	1. Increases cholesterol efflux through the PPARγ-ABCA1/CD36 pathway 2. Upregulated CD36 and AP2 to induce LDL uptake and lipid accumulation	Murine macrophage line RAW264.7 cells	1. Puerarin May increase the ability of M1 macrophages to handle harmful lipids. 2. May support cholesterol homeostasis and exert an anti-atherosclerotic effect.	(201)
	AMI	Reduced apoptosis and oxidative stress damage	Wistar rat MIRI model	1. Puerarin decreased cell necrosis, apoptosis and reduced infarct size. 2. Inhibited cardiotoxicity to ameliorate myocardial infarction.	(203)
	Cardiac dysfunction and myocardial injury	Reduced TNF-α and MDA	SD rat RIRI model	Puerarin improved cardiac contractility and attenuated myocardial and renal injury.	(204)
	MIRI	1. Inhibited caspase-3 activity, upregulated the Bcl-2/Bax ratio, inhibited MPTP over-opening 2. Increased P62, LC3II/I, NDUFB8 and UQCRC2 expression 3.Upregulated the p-AMPK/AMPK ratio 4. Increased HES1 expression.	H9c2 cell A/R injury model	1. Cur pretreatment inhibited ferroptosis, autophagy overactivation and oxidative stress. 2. Improved mitochondrial dysfunction.3. Attenuated apoptosis	(205)
	MIRI	1. Regulated Sirt1/AKT/FoxO3a signaling	SD rat MIRI model/H9c2 cell A/R injury model	Curcumin attenuated MIRI-induced ferroptosis, apoptosis and autophagy.	(8)
Ceria nanozyme coordination with curcumin	Sepsis-induced cardiac injury	1. Inhibited ferroptosis and inflammation 2. Reduced oxidative damage	C57BL/6 mouse sepsis-induced cardiac injury model/H9c2 cells/RAW264.7 cells	Ceria nanozyme coordination with curcumin for treatment of sepsis-induced cardiac injury	(206)
Curcumin	Thoracic aortic aneurysm (TAA)	1. Suppressed of VEGF expression 2. suppressed inflammatory factor expression	Wistar rat TAA model/Patients with degenerative aortic aneurysms	Curcumin attenuates the development of thoracic aortic aneurysm.	(207)
Astragaloside IV	AS	Down-regulated MAPK/NF-κB signaling pathway	LDLR^−/−^ mouse AS model	1. AS-IV decreased the levels of serum lipids, reduced plaque area and increased plaque stability in HFD-induced LDLR^−/−^ mouse. 2. Decreased the levels of inflammatory cytokines	(212)
	AMI	Inhibited the TLR4/MyD88/NF-κB signaling pathway	SD rat AMI model	Alleviate acute myocardial infarction	(213)
	MIRI	Inhibited the STING pathway	C57BL/6 mouse MIRI model/HL1 cell hyposia reoxygenation model	1. Ta-IIA and As-IV alleviates myocardium injury in MIRI. 2. Ta-IIA and As-IV reduced myocardial cell apoptosis, oxidative stress and inflammation.	(214)
	HF	Decreasing the expression of plasma inflammatory markers GDF15, CRP, IL1RL1, and MCP-1, NLRP3, IL-1β, Caspase-1, and IL-6	C57BL/6N mouse HF model	Astragaloside IV alleviates inflammation and improves myocardial metabolism in HFpEF mouse.	(215)
	DCM	Downregulated CD36-mediated ferroptosis	SD rat diabetes model/H9c2 cells	1. Astragaloside IV ameliorated myocardial injury and improved contractile function, attenuated lipid deposition. 2. Astragaloside IV decreased CD36 expression and inhibited lipid accumulation and ferroptosis in PA-induced cardiomyocytes.	(216)
	AAA	1. Abrogated nuclear factor-κB (NF-κB) activation and oxidative stress 2. Inhibited Bap-induced RAW264.7 macrophage cells activation by inhibiting oxidative stress and NF-κB activation through increased phosphorylation of phosphatidylinositol 3-kinase (PI3-K)/AKT	C57/B6J mouse AAA model/RAW264.7 cells	Decreased AAA formation, and reduced macrophage infiltration and expression of matrix metalloproteinase	(217)
Resveratrol	AS	Inhibited macrophage ferroptosis, Alleviated lipid accumulation and inflammation	ApoE^−/−^ mouse AS model/RAW264.7 cells, endothelial cell (EC) line and vascular smooth muscle cell (VSMC) line	Pitavastatin and resveratrol bio-nanocomplexes against hyperhomocysteinemia-induced atherosclerosis.	(218)
	MI	Inhibited ferroptosis via inducing KAT5/GPX4	SD rat MI model/oxygen-glucose deprivation (OGD)-induced H9c2 cardiomyocyte injury model	1. Resveratrol relieves myocardial injury in MI rats. 2. Resveratrol alleviates inflammation and ferroptosis in MI rats.	(219)
	MIRI	Reduced oxidative stress and attenuated ferroptosis	SD rat MIRI model/OGD/R H9c2 cell model	Resveratrol protects against MIRI.	(220)
	MIRI	Inhibited of the VDAC1/GPX4 pathway	C57BL/six mouse MIRI model/A/R H9c2 cell injury model	1. Resveratrol pretreatment effectively attenuated MIRI. 2. Resveratrol pretreatment inhibited MIRI-induced ferroptosis.	(221)
	HF	1. Activated the Sirt1/p53 pathway 2. Inhibited ferroptosis	Sirt1 knockout C57BL/6J mouse in aortic coarctation heart failure model. Using isoproterenol to establish HiPSC cell model.	Resveratrol improved cardiac function in mouse and decelerated ferroptosis and fibrosis progression in heart failure.	(222)
	AS	Mediated up-regulation of GSH	ApoE^−/−^ mouse (Ang)-II-induced AS model/THP-1 cells	Resveratrol attenuate atherosclerosis at least, in part, by inhibiting monocyte differentiation and pro-inflammatory cytokines production.	(223)
	MIRI	1. Inhibited TLR4/NF-κB signaling. 2. Inhibited neutrophil infiltration and TNF- α production	SD rat MIRI model	Resveratrol attenuates the inflammatory reaction induced by I/R injury.	(224)
	HF	Moderated the inflammatory processes in patients with HFrEF	NYHA class II-III HFrEF patients	Resveratrol improved several parameters of heart function, exercise tolerance and quality of life.	(225)
	AAA	Upregulated HMOX1 to reduced extracellular matrix degradation, apoptosis, autophagy, and Inflammation	SD rat AAA model/VSMCs were induced by Ang II to construct the microenvironment of AAA.	1. Resveratrol ameliorated Ang II-induced VSMC dysfunction. 2. Resveratrol suppressed the development of AAA.	(226)

OGD, oxygen-glucose deprivation; NYHA, New York Heart Association; WKY, Wistar–Kyoto; EPCs, endothelial progenitor cells; BMDM, Bone marrow-derived macrophages; HACI, high-altitude cardiac injury; CFs, cardiac fibroblasts; DIC, doxorubicin induced cardiotoxicity; H/R, hypoxia/reoxygenation; A/R, anoxia/reoxygenation; TAA, thoracic aortic aneurysm; HL1, the mouse cardiac muscle cell line; RIRI, renal ischemia-reperfusion injury; HF, heart failure.

## 6 Discussion and future research directions

Ferroptosis, immune-mediated inflammation, and their intricate interplay are increasingly recognized as central drivers of diverse pathologies. This review comprehensively examines dynamic crosstalk in the context of CVDs. We synthesized the current knowledge on the underlying mechanisms, key signaling pathways, and immunomodulatory effects of specific CVDs, including AS, MI, MIRI, HF, and arrhythmias. Furthermore, we critically evaluate emerging therapeutic strategies, such as iron chelators, antioxidants, inflammatory modulators, small-molecule inhibitors, and natural compounds, targeting this axis. This synthesis provides a foundational framework for understanding the pathophysiology and advancing the treatment of ferroptosis- and inflammation-driven CVDs.

The interplay between ferroptosis and immune-mediated inflammation holds considerable promise for CVD treatment; however, several challenges remain. The precise molecular mechanisms underlying ferroptosis have not yet been fully elucidated, particularly across different CVD subtypes, in which ferroptosis may operate via distinct pathways (30, 64).

Immune-mediated inflammation is a critical driver of disease pathogenesis. Key immune cells, including macrophages, DCs, NK cells, and Th17/Treg subsets, orchestrate the initiation, amplification, and resolution of inflammatory responses, primarily through core signaling pathways, such as NF-κB, the NLRP3 inflammasome, and cGAS–STING. Among these, DCs serve a bridging role, and the balance of Th17/Treg cells is central to regulating immune inflammation. This review highlights the pivotal roles of immune cells and inflammatory pathways in cardiovascular pathology. From macrophage polarization imbalance and NLRP3 inflammasome activation to T cell subset dysregulation, these factors collectively form the pathological basis for cardiovascular inflammation.

Nonetheless, key questions regarding immune-mediated mechanisms in the cardiovascular microenvironment remain unresolved: **1)** How do mitochondrial stress and neutrophil extracellular traps (NETs) specifically regulate the activation of the cGAS–STING pathway and its downstream type I interferon response at different stages of AS? 2) How do injury-related molecular patterns precisely control spatiotemporal dynamics of macrophage polarization? 3) Beyond the Th1/Treg imbalance, do metabolic interactions between resident immune cells (e.g., cardiac macrophages) and circulating immune cells determine the ultimate outcome of myocardial repair versus fibrosis?

Furthermore, the bidirectional regulatory roles of different B cell subsets in cardiovascular inflammation are unclear. In-depth exploration of these questions will provide new directions for developing immune-targeted interventions for diseases such as AS and myocardial fibrosis.

However, several issues remain controversial. First, experimental animal models are not sufficiently refined and have inherent limitations. Current model generation methods, such as drug induction and gene knockout, may not fully replicate the multifactorial pathogenesis of human diseases. Moreover, species-specific differences in pathological characteristics, disease progression, genetics, metabolism, and immune responses limit the ability of these models to reflect the true complexity observed in human patients, complicating the translation of these findings into clinical practice. Second, the clinical efficacy of ferroptosis inhibitors, modulators of immune-mediated inflammatory responses, antioxidant defense enhancers, small-molecule inhibitors, traditional Chinese medicines, and their monomers in treating CVDs requires further validation through randomized controlled trials and large-scale multicenter double-blind studies (227, 228). Moreover, the integration of ferroptosis modulation with immunoregulatory strategies to develop cohesive therapeutic regimens represents a critical avenue for future research. Notably, this may include: 1) Multi-omics integration: Constructing single-cell maps of ferroptosis-immunomodulation (e.g., transcriptomic and proteomic profiling within atherosclerotic plaque microenvironments) to elucidate mechanistic links and identify novel therapeutic targets for AS, MI, and related disorders. 2) Closed-loop diagnostic and therapeutic system: Leveraging real-time monitoring of serum ferroptosis markers and inflammatory biomarkers, such as IL-18, combined with artificial intelligence algorithms and individualized treatment plans to enable precision therapy. For instance, bionic nanoparticles (CD47+) loaded with Fer-1 can be engineered to target MI sites and mitigate cardiomyocyte ferroptosis.

The concept of personalized medicine should be more effectively implemented in CVD management to deliver patient-specific interventions. Genetic research is likely to illuminate new directions and controversies regarding ferroptosis-driven inflammation. For example, a 2024 large-scale genome-wide association study (Eur Heart J) identified the GPX4 locus (rs713041) as significantly associated with coronary artery disease risk (odds ratio: 1.32, P = 3.1 × 10^−8^), suggesting that GPX4 variants may elevate coronary heart disease risk via ferroptosis modulation (229). Furthermore, a dose–response relationship has been observed between iron metabolism-related genes (e.g., TFR2 rs7385804) and circulating levels of the inflammatory marker IL-6, supporting a mechanistic link between iron homeostasis and inflammation.

However, some evidence challenges the notion that ferroptosis directly drives inflammatory responses. Although ALOX15 polymorphisms influence LPO, they show no direct association with NLRP3 inflammasome activation (P = 0.17), implying that LPO may modulate inflammation through alternative pathways. Additionally, in populations of African descent, no co-localization was observed between specific FTH1 gene variants and inflammatory markers, suggesting potential population-specific differences in how iron metabolism genes influence inflammatory processes (229).

Despite these unresolved issues, the combined modulation of ferroptosis and immune responses remains a promising strategy for treating CVD. Future research should strengthen the theoretical framework and provide practical guidance for precision cardiovascular therapeutics.

## 7 Conclusions

The interplay between ferroptosis and immune-mediated inflammatory responses has garnered increasing attention in CVD and MI research. As our understanding of their roles in disease progression deepens, it has become evident that these processes are not only independent pathological mechanisms but also components of a highly intertwined and complex network. Ferroptosis, a recently characterized form of regulated cell death, primarily induces cell death through iron overload and LPO, while immune-mediated inflammation further amplifies tissue damage. The crosstalk between these two processes may play a pivotal role in the pathogenesis of CVD, offering novel avenues for research and potential therapeutic interventions in the future. Through a comprehensive investigation in this field, new strategies for the prevention and treatment of CVDs are likely to emerge, ultimately improving patient quality of life and clinical outcomes. Therefore, continuous monitoring of advances in ferroptosis and immune modulation is essential to facilitate the integration of basic research with clinical applications and address the increasingly complex challenges posed by CVDs.

## References

[1] Boczar KE Beanlands R Wells G Coyle D . Cost-effectiveness of canakinumab from a Canadian perspective for recurrent cardiovascular events. CJC Open. (2022) 4:441–8. doi: 10.1016/j.cjco.2022.01.003, PMID: 35607490 PMC9123368

[2] Wohlford GF Van Tassell BW Billingsley HE Kadariya D Canada JM Carbone S . Phase 1b, randomized, double-blinded, dose escalation, single-center, repeat dose safety and pharmacodynamics study of the oral Nlrp3 inhibitor dapansutrile in subjects with nyha ii-iii systolic heart failure. J Cardiovasc Pharmacol. (2020) 77:49–60. doi: 10.1097/fjc.0000000000000931, PMID: 33235030 PMC7774821

[3] Zeng Q Jiang T . Molecular mechanisms of ferroptosis in cardiovascular disease. Mol Cell Biochem. (2024) 479:3181–93. doi: 10.1007/s11010-024-04940-2, PMID: 38374233

[4] Xu X Xu XD Ma MQ Liang Y Cai YB Zhu ZX . The mechanisms of ferroptosis and its role in atherosclerosis. BioMed Pharmacother. (2024) 171:116112. doi: 10.1016/j.biopha.2023.116112, PMID: 38171246

[5] Wang XD Kang S . Ferroptosis in myocardial infarction: not a marker but a maker. Open Biol. (2021) 11:200367. doi: 10.1098/rsob.200367, PMID: 33878951 PMC8059645

[6] Zhao WK Zhou Y Xu TT Wu Q . Ferroptosis: opportunities and challenges in myocardial ischemia-reperfusion injury. Oxid Med Cell Longev. (2021) 2021:9929687. doi: 10.1155/2021/9929687, PMID: 34725566 PMC8557044

[7] Hu H Chen Y Jing L Zhai C Shen L . The link between ferroptosis and cardiovascular diseases: A novel target for treatment. Front Cardiovasc Med. (2021) 8:710963. doi: 10.3389/fcvm.2021.710963, PMID: 34368260 PMC8341300

[8] Zhao ST Qiu ZC Xu ZQ Tao ED Qiu RB Peng HZ . Curcumin attenuates myocardial ischemia−Reperfusion−Induced autophagy−Dependent ferroptosis via Sirt1/Akt/Foxo3a signaling. Int J Mol Med. (2025) 55(3):51. doi: 10.3892/ijmm.2025.5492, PMID: 39930816 PMC11781526

[9] Li W Li W Leng Y Xiong Y Xia Z . Ferroptosis is involved in diabetes myocardial ischemia/reperfusion injury through endoplasmic reticulum stress. DNA Cell Biol. (2020) 39:210–25. doi: 10.1089/dna.2019.5097, PMID: 31809190

[10] Fang X Wang H Han D Xie E Yang X Wei J . Ferroptosis as a target for protection against cardiomyopathy. Proc Natl Acad Sci U.S.A. (2019) 116:2672–80. doi: 10.1073/pnas.1821022116, PMID: 30692261 PMC6377499

[11] Tadokoro T Ikeda M Ide T Deguchi H Ikeda S Okabe K . Mitochondria-dependent ferroptosis plays a pivotal role in doxorubicin cardiotoxicity. JCI Insight. (2020) 5(9):e132747. doi: 10.1172/jci.insight.132747, PMID: 32376803 PMC7253028

[12] Wang X Chen X Zhou W Men H Bao T Sun Y . Ferroptosis is essential for diabetic cardiomyopathy and is prevented by sulforaphane via Ampk/Nrf2 pathways. Acta Pharm Sin B. (2022) 12:708–22. doi: 10.1016/j.apsb.2021.10.005, PMID: 35256941 PMC8897044

[13] Wang C Yuan W Hu A Lin J Xia Z Yang CF . Dexmedetomidine alleviated sepsis−Induced myocardial ferroptosis and septic heart injury. Mol Med Rep. (2020) 22:175–84. doi: 10.3892/mmr.2020.11114, PMID: 32377745 PMC7248514

[14] Dai C Kong B Qin T Xiao Z Fang J Gong Y . Inhibition of ferroptosis reduces susceptibility to frequent excessive alcohol consumption-induced atrial fibrillation. Toxicology. (2022) 465:153055. doi: 10.1016/j.tox.2021.153055, PMID: 34864093

[15] Chen P Li X . Nlrp3 inflammasome in atherosclerosis: mechanisms and targeted therapies. Front Pharmacol. (2024) 15:1430236. doi: 10.3389/fphar.2024.1430236, PMID: 39144618 PMC11322363

[16] Yang Y Wang Y Guo L Gao W Tang TL Yan M . Interaction between macrophages and ferroptosis. Cell Death Dis. (2022) 13:355. doi: 10.1038/s41419-022-04775-z, PMID: 35429990 PMC9013379

[17] Chen X Kang R Kroemer G Tang D . Ferroptosis in infection, inflammation, and immunity. J Exp Med. (2021) 218(6):e20210518. doi: 10.1084/jem.20210518, PMID: 33978684 PMC8126980

[18] Ma J Li C Liu T Zhang L Wen X Liu X . Identification of markers for diagnosis and treatment of diabetic kidney disease based on the ferroptosis and immune. Oxid Med Cell Longev. (2022) 2022:9957172. doi: 10.1155/2022/9957172, PMID: 36466094 PMC9712001

[19] Dixon SJ Lemberg KM Lamprecht MR Skouta R Zaitsev EM Gleason CE . Ferroptosis: an iron-dependent form of nonapoptotic cell death. Cell. (2012) 149:1060–72. doi: 10.1016/j.cell.2012.03.042, PMID: 22632970 PMC3367386

[20] Ramos S Hartenian E Santos JC Walch P Broz P . Ninj1 induces plasma membrane rupture and release of damage-associated molecular pattern molecules during ferroptosis. EMBO J. (2024) 43:1164–86. doi: 10.1038/s44318-024-00055-y, PMID: 38396301 PMC10987646

[21] Wu A Feng B Yu J Yan L Che L Zhuo Y . Fibroblast growth factor 21 attenuates iron overload-induced liver injury and fibrosis by inhibiting ferroptosis. Redox Biol. (2021) 46:102131. doi: 10.1016/j.redox.2021.102131, PMID: 34530349 PMC8445902

[22] Feng X Wang S Sun Z Dong H Yu H Huang M . Ferroptosis enhanced diabetic renal tubular injury via Hif-1α/Ho-1 pathway in Db/Db mice. Front Endocrinol (Lausanne). (2021) 12:626390. doi: 10.3389/fendo.2021.626390, PMID: 33679620 PMC7930496

[23] Bao WD Pang P Zhou XT Hu F Xiong W Chen K . Loss of ferroportin induces memory impairment by promoting ferroptosis in Alzheimer's disease. Cell Death Differ. (2021) 28:1548–62. doi: 10.1038/s41418-020-00685-9, PMID: 33398092 PMC8166828

[24] Zhou Q Meng Y Li D Yao L Le J Liu Y . Ferroptosis in cancer: from molecular mechanisms to therapeutic strategies. Signal Transduct Target Ther. (2024) 9:55. doi: 10.1038/s41392-024-01769-5, PMID: 38453898 PMC10920854

[25] Fan X Li A Yan Z Geng X Lian L Lv H . From iron metabolism to ferroptosis: pathologic changes in coronary heart disease. Oxid Med Cell Longev. (2022) 2022:6291889. doi: 10.1155/2022/6291889, PMID: 35993022 PMC9385341

[26] Sawicki KT De Jesus A Ardehali H . Iron metabolism in cardiovascular disease: physiology, mechanisms, and therapeutic targets. Circ Res. (2023) 132:379–96. doi: 10.1161/circresaha.122.321667, PMID: 36730380 PMC9907000

[27] Wang J Shi H Yang Y Gong X . Crosstalk between ferroptosis and innate immune in diabetic kidney disease: mechanisms and therapeutic implications. Front Immunol. (2025) 16:1505794. doi: 10.3389/fimmu.2025.1505794, PMID: 40092979 PMC11906378

[28] Zhao Y Linkermann A Takahashi M Li Q Zhou X . Ferroptosis in cardiovascular disease: regulatory mechanisms and therapeutic implications. Eur Heart J. (2025) 46:3247–60. doi: 10.1093/eurheartj/ehaf374, PMID: 40464746

[29] Dixon SJ Olzmann JA . The cell biology of ferroptosis. Nat Rev Mol Cell Biol. (2024) 25:424–42. doi: 10.1038/s41580-024-00703-5, PMID: 38366038 PMC12187608

[30] Ru Q Li Y Chen L Wu Y Min J Wang F . Iron homeostasis and ferroptosis in human diseases: mechanisms and therapeutic prospects. Signal Transduct Target Ther. (2024) 9:271. doi: 10.1038/s41392-024-01969-z, PMID: 39396974 PMC11486532

[31] Sun S Shen J Jiang J Wang F Min J . Targeting ferroptosis opens new avenues for the development of novel therapeutics. Signal Transduct Target Ther. (2023) 8:372. doi: 10.1038/s41392-023-01606-1, PMID: 37735472 PMC10514338

[32] Carpenter S O'Neill LAJ . From periphery to center stage: 50 years of advancements in innate immunity. Cell. (2024) 187:2030–51. doi: 10.1016/j.cell.2024.03.036, PMID: 38670064 PMC11060700

[33] Dikiy S Rudensky AY . Principles of regulatory T cell function. Immunity. (2023) 56:240–55. doi: 10.1016/j.immuni.2023.01.004, PMID: 36792571

[34] Liu G Xie X Liao W Chen S Zhong R Qin J . Ferroptosis in cardiovascular disease. BioMed Pharmacother. (2024) 170:116057. doi: 10.1016/j.biopha.2023.116057, PMID: 38159373

[35] Chen R Zhang H Tang B Luo Y Yang Y Zhong X . Macrophages in cardiovascular diseases: molecular mechanisms and therapeutic targets. Signal Transduct Target Ther. (2024) 9:130. doi: 10.1038/s41392-024-01840-1, PMID: 38816371 PMC11139930

[36] Zhang L Li J Kou Y Shen L Wang H Wang Y . Mechanisms and treatment of atherosclerosis: focus on macrophages. Front Immunol. (2024) 15:1490387. doi: 10.3389/fimmu.2024.1490387, PMID: 39569201 PMC11576186

[37] Xu J Han X Xia N Zhao Q Cheng Z . Il−37 suppresses macrophage ferroptosis to attenuate diabetic atherosclerosis via the Nrf2 pathway. Exp Ther Med. (2023) 25:289. doi: 10.3892/etm.2023.11988, PMID: 37206550 PMC10189585

[38] Biasucci LM La Rosa G Pedicino D D'Aiello A Galli M Liuzzo G . Where does inflammation fit? Curr Cardiol Rep. (2017) 19:84. doi: 10.1007/s11886-017-0896-0, PMID: 28779286

[39] Saigusa R Winkels H Ley K . T cell subsets and functions in atherosclerosis. Nat Rev Cardiol. (2020) 17:387–401. doi: 10.1038/s41569-020-0352-5, PMID: 32203286 PMC7872210

[40] Martín P Sánchez-Madrid F . T cells in cardiac health and disease. J Clin Invest. (2025) 135(2):e185218. doi: 10.1172/jci185218, PMID: 39817455 PMC11735099

[41] Li JY Yao YM Tian YP . Ferroptosis: A trigger of proinflammatory state progression to immunogenicity in necroinflammatory disease. Front Immunol. (2021) 12:701163. doi: 10.3389/fimmu.2021.701163, PMID: 34489948 PMC8418153

[42] Xue J Suarez JS Minaai M Li S Gaudino G Pass HI . Hmgb1 as a therapeutic target in disease. J Cell Physiol. (2021) 236:3406–19. doi: 10.1002/jcp.30125, PMID: 33107103 PMC8104204

[43] Zhao Y Wu J Liu X Chen X Wang J . Decoding nature: multi-target anti-inflammatory mechanisms of natural products in the Tlr4/Nf-Kb pathway. Front Pharmacol. (2024) 15:1467193. doi: 10.3389/fphar.2024.1467193, PMID: 39877388 PMC11772364

[44] Zhu K Fan R Cao Y Yang W Zhang Z Zhou Q . Glycyrrhizin attenuates myocardial ischemia reperfusion injury by suppressing inflammation, oxidative stress, and ferroptosis via the Hmgb1-Tlr4-Gpx4 pathway. Exp Cell Res. (2024) 435:113912. doi: 10.1016/j.yexcr.2024.113912, PMID: 38176464

[45] Wen Q Liu J Kang R Zhou B Tang D . The release and activity of Hmgb1 in ferroptosis. Biochem Biophys Res Commun. (2019) 510:278–83. doi: 10.1016/j.bbrc.2019.01.090, PMID: 30686534

[46] Ding HS Huang Y Qu JF Wang YJ Huang ZY Wang FY . Panaxynol ameliorates cardiac ischemia/reperfusion injury by suppressing nlrp3-induced pyroptosis and apoptosis via Hmgb1/Tlr4/Nf-Kb axis. Int Immunopharmacol. (2023) 121:110222. doi: 10.1016/j.intimp.2023.110222, PMID: 37343367

[47] Hacioglu C . Long-term exposure of sucralose induces neuroinflammation and ferroptosis in human microglia cells via Sirt1/Nlrp3/Il-1β/Gpx4 signaling pathways. Food Sci Nutr. (2024) 12:9094–107. doi: 10.1002/fsn3.4488, PMID: 39619997 PMC11606902

[48] Li GZ Liu JY Zhou H . Ferroptosis: A novel therapeutic target for diabetic cardiomyopathy. World J Diabetes. (2025) 16:104665. doi: 10.4239/wjd.v16.i6.104665, PMID: 40548263 PMC12179866

[49] Liang D Minikes AM Jiang X . Ferroptosis at the intersection of lipid metabolism and cellular signaling. Mol Cell. (2022) 82:2215–27. doi: 10.1016/j.molcel.2022.03.022, PMID: 35390277 PMC9233073

[50] Li H Cao Z Wang L Liu C Lin H Tang Y . Macrophage subsets and death are responsible for atherosclerotic plaque formation. Front Immunol. (2022) 13:843712. doi: 10.3389/fimmu.2022.843712, PMID: 35432323 PMC9007036

[51] Wu KK Cheung SW Cheng KK . Nlrp3 inflammasome activation in adipose tissues and its implications on metabolic diseases. Int J Mol Sci. (2020) 21(11):4184. doi: 10.3390/ijms21114184, PMID: 32545355 PMC7312293

[52] Wisitpongpun P Potup P Usuwanthim K . Oleamide-mediated polarization of M1 macrophages and Il-1β Production by regulating Nlrp3-inflammasome activation in primary human monocyte-derived macrophages. Front Immunol. (2022) 13:856296. doi: 10.3389/fimmu.2022.856296, PMID: 35514993 PMC9062104

[53] Dzobo KE Cupido AJ Mol BM Stiekema LCA Versloot M Winkelmeijer M . Diacylglycerols and lysophosphatidic acid, enriched on lipoprotein(a), contribute to monocyte inflammation. Arterioscler Thromb Vasc Biol. (2024) 44:720–40. doi: 10.1161/atvbaha.123.319937, PMID: 38269588 PMC10880937

[54] Linke M Fritsch SD Sukhbaatar N Hengstschläger M Weichhart T . Mtorc1 and Mtorc2 as regulators of cell metabolism in immunity. FEBS Lett. (2017) 591:3089–103. doi: 10.1002/1873-3468.12711, PMID: 28600802 PMC6322652

[55] Li D Wu M . Pattern recognition receptors in health and diseases. Signal Transduct Target Ther. (2021) 6:291. doi: 10.1038/s41392-021-00687-0, PMID: 34344870 PMC8333067

[56] Zindel J Kubes P . Damps, pamps, and lamps in immunity and sterile inflammation. Annu Rev Pathol. (2020) 15:493–518. doi: 10.1146/annurev-pathmechdis-012419-032847, PMID: 31675482

[57] Gong T Liu L Jiang W Zhou R . Damp-sensing receptors in sterile inflammation and inflammatory diseases. Nat Rev Immunol. (2020) 20:95–112. doi: 10.1038/s41577-019-0215-7, PMID: 31558839

[58] Ueda N Takasawa K . Impact of inflammation on ferritin, hepcidin and the management of iron deficiency anemia in chronic kidney disease. Nutrients. (2018) 10(9):1173. doi: 10.3390/nu10091173, PMID: 30150549 PMC6163440

[59] Pérez S Rius-Pérez S . Macrophage polarization and reprogramming in acute inflammation: A redox perspective. Antioxidants (Basel). (2022) 11(7):1394. doi: 10.3390/antiox11071394, PMID: 35883885 PMC9311967

[60] Wang L He C . Nrf2-mediated anti-inflammatory polarization of macrophages as therapeutic targets for osteoarthritis. Front Immunol. (2022) 13:967193. doi: 10.3389/fimmu.2022.967193, PMID: 36032081 PMC9411667

[61] Ni S Yuan Y Kuang Y Li X . Iron metabolism and immune regulation. Front Immunol. (2022) 13:816282. doi: 10.3389/fimmu.2022.816282, PMID: 35401569 PMC8983924

[62] Wu P Chen J Li H Lu H Li Y Zhang J . Interactions between ferroptosis and tumour development mechanisms: implications for gynaecological cancer therapy (Review). Oncol Rep. (2025) 53(2):18. doi: 10.3892/or.2024.8851, PMID: 39635847 PMC11638741

[63] Jiang Q Wan R Jiang J Li T Li Y Yu S . Interaction between macrophages and ferroptosis: metabolism, function, and diseases. Chin Med J (Engl). (2025) 138:509–22. doi: 10.1097/cm9.0000000000003189, PMID: 39245648 PMC11882282

[64] Zhang J Guo C . Current progress of ferroptosis in cardiovascular diseases. Front Cardiovasc Med. (2023) 10:1259219. doi: 10.3389/fcvm.2023.1259219, PMID: 37942067 PMC10628442

[65] Yu X Zhu D Luo B Kou W Cheng Y Zhu Y . Ifnγ Enhances ferroptosis by increasing Jak−Stat pathway activation to suppress Slca711 expression in adrenocortical carcinoma. Oncol Rep. (2022) 47(5):97. doi: 10.3892/or.2022.8308, PMID: 35322867 PMC8968764

[66] Dai Y Cui C Jiao D Zhu X . Jak/Stat signaling as a key regulator of ferroptosis: mechanisms and therapeutic potentials in cancer and diseases. Cancer Cell Int. (2025) 25:83. doi: 10.1186/s12935-025-03681-6, PMID: 40055704 PMC11889932

[67] Wu W Luo Z Shen D Lan T Xiao Z Liu M . Il-10 protects against Opc ferroptosis by regulating lipid reactive oxygen species levels post stroke. Redox Biol. (2024) 69:102982. doi: 10.1016/j.redox.2023.102982, PMID: 38070317 PMC10755589

[68] Ma XH Liu JH Liu CY Sun WY Duan WJ Wang G . Alox15-launched pufa-phospholipids peroxidation increases the susceptibility of ferroptosis in ischemia-induced myocardial damage. Signal Transduct Target Ther. (2022) 7:288. doi: 10.1038/s41392-022-01090-z, PMID: 35970840 PMC9378747

[69] Hutchins AP Diez D Miranda-Saavedra D . The Il-10/Stat3-mediated anti-inflammatory response: recent developments and future challenges. Brief Funct Genomics. (2013) 12:489–98. doi: 10.1093/bfgp/elt028, PMID: 23943603 PMC3838198

[70] Huang Y Xu W Zhou R . Nlrp3 inflammasome activation and cell death. Cell Mol Immunol. (2021) 18:2114–27. doi: 10.1038/s41423-021-00740-6, PMID: 34321623 PMC8429580

[71] Kelley N Jeltema D Duan Y He Y . The Nlrp3 inflammasome: an overview of mechanisms of activation and regulation. Int J Mol Sci. (2019) 20(13):3328. doi: 10.3390/ijms20133328, PMID: 31284572 PMC6651423

[72] Feng S Wierzbowski MC Hrovat-Schaale K Dumortier A Zhang Y Zyulina M . Mechanisms of Nlrp3 activation and inhibition elucidated by functional analysis of disease-associated variants. Nat Immunol. (2025) 26:511–23. doi: 10.1038/s41590-025-02088-9, PMID: 39930093 PMC11876074

[73] Blevins HM Xu Y Biby S Zhang S . The Nlrp3 inflammasome pathway: A review of mechanisms and inhibitors for the treatment of inflammatory diseases. Front Aging Neurosci. (2022) 14:879021. doi: 10.3389/fnagi.2022.879021, PMID: 35754962 PMC9226403

[74] Lin Y Sheng M Qin H Zhang P Wang C Fu W . Caspase 6 promotes innate immune activation by functional crosstalk between Ripk1-Iκbα Axis in liver inflammation. Cell Commun Signal. (2023) 21:282. doi: 10.1186/s12964-023-01287-x, PMID: 37828624 PMC10568785

[75] Wu X Wei J Yi Y Gong Q Gao J . Activation of Nrf2 signaling: A key molecular mechanism of protection against cardiovascular diseases by natural products. Front Pharmacol. (2022) 13:1057918. doi: 10.3389/fphar.2022.1057918, PMID: 36569290 PMC9772885

[76] Yang R Gao W Wang Z Jian H Peng L Yu X . Polyphyllin I induced ferroptosis to suppress the progression of hepatocellular carcinoma through activation of the mitochondrial dysfunction via Nrf2/Ho-1/Gpx4 axis. Phytomedicine. (2024) 122:155135. doi: 10.1016/j.phymed.2023.155135, PMID: 37856990

[77] Xiong M Luo R Zhang Z Liu P Peng Q Xu F . Il-27 regulates macrophage ferroptosis by inhibiting the Nrf2/Ho1 signaling pathway in sepsis-induced Ards. Inflammation Res. (2025) 74:39. doi: 10.1007/s00011-024-01986-2, PMID: 39945893

[78] Liu W Tan Z Zhao Y Zhao Y Yu X Wang B . Panaxadiol saponin ameliorates ferroptosis in iron-overload aplastic anemia mice and Meg-01 cells by activating Nrf2/Ho-1 and Pi3k/Akt/Mtor signaling pathway. Int Immunopharmacol. (2023) 118:110131. doi: 10.1016/j.intimp.2023.110131, PMID: 37023700

[79] Zhang Y Ye P Zhu H Gu L Li Y Feng S . Neutral polysaccharide from gastrodia elata alleviates cerebral ischemia-reperfusion injury by inhibiting ferroptosis-mediated neuroinflammation via the Nrf2/Ho-1 signaling pathway. CNS Neurosci Ther. (2024) 30:e14456. doi: 10.1111/cns.14456, PMID: 37752806 PMC10916450

[80] Wu S Zhu J Wu G Hu Z Ying P Bao Z . 6-gingerol alleviates ferroptosis and inflammation of diabetic cardiomyopathy via the Nrf2/Ho-1 pathway. Oxid Med Cell Longev. (2022) 2022:3027514. doi: 10.1155/2022/3027514, PMID: 36624878 PMC9825225

[81] Bouchaoui H Mahoney-Sanchez L Garçon G Berdeaux O Alleman LY Devos D . Acsl4 and the lipoxygenases 15/15b are pivotal for ferroptosis induced by iron and pufa dyshomeostasis in dopaminergic neurons. Free Radic Biol Med. (2023) 195:145–57. doi: 10.1016/j.freeradbiomed.2022.12.086, PMID: 36581060

[82] Lee JY Kim WK Bae KH Lee SC Lee EW . Lipid metabolism and ferroptosis. Biol (Basel). (2021) 10(3):184. doi: 10.3390/biology10030184, PMID: 33801564 PMC8000263

[83] Kim JW Lee JY Oh M Lee EW . An integrated view of lipid metabolism in ferroptosis revisited via lipidomic analysis. Exp Mol Med. (2023) 55:1620–31. doi: 10.1038/s12276-023-01077-y, PMID: 37612411 PMC10474074

[84] Cai W Liu L Shi X Liu Y Wang J Fang X . Alox15/15-hpete aggravates myocardial ischemia-reperfusion injury by promoting cardiomyocyte ferroptosis. Circulation. (2023) 147:1444–60. doi: 10.1161/circulationaha.122.060257, PMID: 36987924

[85] Zhang Y Men J Yin K Zhang Y Yang J Li X . Activation of gut metabolite Acsl4/Lpcat3 by microplastics in drinking water mediates ferroptosis via gut-kidney axis. Commun Biol. (2025) 8:211. doi: 10.1038/s42003-025-07641-8, PMID: 39930042 PMC11811008

[86] Yang J Shi X Wang Y Ma M Liu H Wang J . Multi-target neuroprotection of thiazolidinediones on Alzheimer's disease via neuroinflammation and ferroptosis. J Alzheimers Dis. (2023) 96:927–45. doi: 10.3233/jad-230593, PMID: 37927258 PMC10741341

[87] Zhang Q Lenardo MJ Baltimore D . 30 years of Nf-Kb: A blossoming of relevance to human pathobiology. Cell. (2017) 168:37–57. doi: 10.1016/j.cell.2016.12.012, PMID: 28086098 PMC5268070

[88] Yu H Lin L Zhang Z Zhang H Hu H . Targeting Nf-Kb pathway for the therapy of diseases: mechanism and clinical study. Signal Transduct Target Ther. (2020) 5:209. doi: 10.1038/s41392-020-00312-6, PMID: 32958760 PMC7506548

[89] Mitchell JP Carmody RJ . Nf-Kb and the transcriptional control of inflammation. Int Rev Cell Mol Biol. (2018) 335:41–84. doi: 10.1016/bs.ircmb.2017.07.007, PMID: 29305014

[90] Chen Y Fang ZM Yi X Wei X Jiang DS . The interaction between ferroptosis and inflammatory signaling pathways. Cell Death Dis. (2023) 14:205. doi: 10.1038/s41419-023-05716-0, PMID: 36944609 PMC10030804

[91] Wu S Zhou Y Liang J Ying P Situ Q Tan X . Upregulation of Nf-Kb by Usp24 aggravates ferroptosis in diabetic cardiomyopathy. Free Radic Biol Med. (2024) 210:352–66. doi: 10.1016/j.freeradbiomed.2023.11.032, PMID: 38056575

[92] Yan N Xu Z Qu C Zhang J . Dimethyl fumarate improves cognitive deficits in chronic cerebral hypoperfusion rats by alleviating inflammation, oxidative stress, and ferroptosis via Nrf2/Are/Nf-Kb signal pathway. Int Immunopharmacol. (2021) 98:107844. doi: 10.1016/j.intimp.2021.107844, PMID: 34153667

[93] Xiao Z Kong B Fang J Qin T Dai C Shuai W . Ferrostatin-1 alleviates lipopolysaccharide-induced cardiac dysfunction. Bioengineered. (2021) 12:9367–76. doi: 10.1080/21655979.2021.2001913, PMID: 34787054 PMC8809987

[94] Xue C Yao Q Gu X Shi Q Yuan X Chu Q . Evolving cognition of the Jak-Stat signaling pathway: autoimmune disorders and cancer. Signal Transduct Target Ther. (2023) 8:204. doi: 10.1038/s41392-023-01468-7, PMID: 37208335 PMC10196327

[95] Philips RL Wang Y Cheon H Kanno Y Gadina M Sartorelli V . The Jak-Stat pathway at 30: much learned, much more to do. Cell. (2022) 185:3857–76. doi: 10.1016/j.cell.2022.09.023, PMID: 36240739 PMC9815833

[96] Barrat FJ Crow MK Ivashkiv LB . Interferon target-gene expression and epigenomic signatures in health and disease. Nat Immunol. (2019) 20:1574–83. doi: 10.1038/s41590-019-0466-2, PMID: 31745335 PMC7024546

[97] Kong R Wang N Han W Bao W Lu J . Ifnγ-mediated repression of system Xc(-) drives vulnerability to induced ferroptosis in hepatocellular carcinoma cells. J Leukoc Biol. (2021) 110:301–14. doi: 10.1002/jlb.3ma1220-815rrr, PMID: 34318944

[98] Maliken BD Nelson JE Kowdley KV . The hepcidin circuits act: balancing iron and inflammation. Hepatology. (2011) 53:1764–6. doi: 10.1002/hep.24267, PMID: 21520181 PMC3095435

[99] Poindessous V Lazareth H Crambert G Cheval L Sampaio JL Pallet N . Stat3 drives the expression of Acsl4 in acute kidney injury. iScience. (2024) 27:109737. doi: 10.1016/j.isci.2024.109737, PMID: 38799564 PMC11126884

[100] Yang L Wang H Yang X Wu Q An P Jin X . Auranofin mitigates systemic iron overload and induces ferroptosis via distinct mechanisms. Signal Transduct Target Ther. (2020) 5:138. doi: 10.1038/s41392-020-00253-0, PMID: 32732975 PMC7393508

[101] Al-Rasheed NM Al-Rasheed NM Hasan IH Al-Amin MA Al-Ajmi HN Mahmoud AM . Sitagliptin attenuates cardiomyopathy by modulating the Jak/Stat signaling pathway in experimental diabetic rats. Drug Des Devel Ther. (2016) 10:2095–107. doi: 10.2147/dddt.S109287, PMID: 27418808 PMC4933570

[102] Zhang X He J Xu Z Yang Y . Naringin regulates the Cgas-sting pathway to improve mitochondrial dysfunction and ferroptosis after myocardial ischemia-reperfusion injury. Cytotechnology. (2025) 77:103. doi: 10.1007/s10616-025-00762-2, PMID: 40384845 PMC12084444

[103] Wang X Tan X Zhang J Wu J Shi H . The emerging roles of Mapk-Ampk in ferroptosis regulatory network. Cell Commun Signal. (2023) 21:200. doi: 10.1186/s12964-023-01170-9, PMID: 37580745 PMC10424420

[104] Chen W Zhang Y Wang Z Tan M Lin J Qian X . Dapagliflozin alleviates myocardial ischemia/reperfusion injury by reducing ferroptosis via Mapk signaling inhibition. Front Pharmacol. (2023) 14:1078205. doi: 10.3389/fphar.2023.1078205, PMID: 36891270 PMC9986553

[105] Bai B Ji Z Wang F Qin C Zhou H Li D . Ctrp12 ameliorates post-myocardial infarction heart failure through down-regulation of cardiac apoptosis, oxidative stress and inflammation by influencing the Tak1-P38 Mapk/Jnk pathway. Inflammation Res. (2023) 72:1375–90. doi: 10.1007/s00011-023-01758-4, PMID: 37382682

[106] Yang Z He Y Wu D Shi W Liu P Tan J . Antiferroptosis therapy alleviated the development of atherosclerosis. MedComm (2020). (2024) 5:e520. doi: 10.1002/mco2.520, PMID: 38576455 PMC10993356

[107] Bai T Li M Liu Y Qiao Z Wang Z . Inhibition of ferroptosis alleviates atherosclerosis through attenuating lipid peroxidation and endothelial dysfunction in mouse aortic endothelial cell. Free Radic Biol Med. (2020) 160:92–102. doi: 10.1016/j.freeradbiomed.2020.07.026, PMID: 32768568

[108] Li Y Ma JQ Wang CC Zhou J Sun YD Wei XL . Ferroptosis: A potential target of macrophages in plaque vulnerability. Open Life Sci. (2023) 18:20220722. doi: 10.1515/biol-2022-0722, PMID: 37791060 PMC10543703

[109] Li Y Zhang L Zhang Q Zhang Y Pan S Zhao H . Hspb1 suppresses oxldl-induced vascular smooth muscle cell ferroptosis by inhibiting Dpp4. Arch Biochem Biophys. (2025) 768:110400. doi: 10.1016/j.abb.2025.110400, PMID: 40132776

[110] Xiang P Chen Q Chen L Lei J Yuan Z Hu H . Metabolite neu5ac triggers Slc3a2 degradation promoting vascular endothelial ferroptosis and aggravates atherosclerosis progression in Apoe(-/-)Mice. Theranostics. (2023) 13:4993–5016. doi: 10.7150/thno.87968, PMID: 37771765 PMC10526676

[111] Lu X Ji Q Pan H Feng Y Ye D Gan L . Il-23p19 deficiency reduces M1 macrophage polarization and improves stress-induced cardiac remodeling by alleviating macrophage ferroptosis in mice. Biochem Pharmacol. (2024) 222:116072. doi: 10.1016/j.bcp.2024.116072, PMID: 38387530

[112] Gao M Dong L Yang Y Yan J Liang Y Ma X . The anti-atherosclerotic effect of paeonol against the lipid accumulation in macrophage-derived foam cells by inhibiting ferroptosis via the Sirt1/Nrf2/Gpx4 signaling pathway. Biochem Biophys Res Commun. (2024) 708:149788. doi: 10.1016/j.bbrc.2024.149788, PMID: 38518720

[113] Gao X Hu W Qian D Bai X He H Li L . The mechanisms of ferroptosis under hypoxia. Cell Mol Neurobiol. (2023) 43:3329–41. doi: 10.1007/s10571-023-01388-8, PMID: 37458878 PMC10477166

[114] Yu D Hu Y Ma M Li W Zhao X . The landscape of research on ferroptosis under hypoxic conditions: A bibliometric analysis. Front Pharmacol. (2025) 16:1519000. doi: 10.3389/fphar.2025.1519000, PMID: 40206079 PMC11979267

[115] Wang K Chen XZ Wang YH Cheng XL Zhao Y Zhou LY . Emerging roles of ferroptosis in cardiovascular diseases. Cell Death Discov. (2022) 8:394. doi: 10.1038/s41420-022-01183-2, PMID: 36127318 PMC9488879

[116] Qian Y Chen L Gao B Ye X . Sestrin2 levels in patients with anxiety and depression myocardial infarction was up-regulated and suppressed inflammation and ferroptosis by Lkb1-mediated Ampk activation. Clin Exp Hypertens. (2023) 45:2205049. doi: 10.1080/10641963.2023.2205049, PMID: 37183711

[117] Jin B Zhang Z Zhang Y Yang M Wang C Xu J . Ferroptosis and myocardial ischemia-reperfusion: mechanistic insights and new therapeutic perspectives. Front Pharmacol. (2024) 15:1482986. doi: 10.3389/fphar.2024.1482986, PMID: 39411064 PMC11473306

[118] Ge Y Huang M Yao YM . The effect and regulatory mechanism of high mobility group Box-1 protein on immune cells in inflammatory diseases. Cells. (2021) 10(5):1044. doi: 10.3390/cells10051044, PMID: 33925132 PMC8145631

[119] Chen R Kang R Tang D . The mechanism of Hmgb1 secretion and release. Exp Mol Med. (2022) 54:91–102. doi: 10.1038/s12276-022-00736-w, PMID: 35217834 PMC8894452

[120] Zhao K Chen X Bian Y Zhou Z Wei X Zhang J . Broadening horizons: the role of ferroptosis in myocardial ischemia-reperfusion injury. Naunyn Schmiedebergs Arch Pharmacol. (2023) 396:2269–86. doi: 10.1007/s00210-023-02506-5, PMID: 37119287

[121] Perticone M Gigliotti S Shehaj E Maio R Suraci E Miceli S . Gut permeability and immune-mediated inflammation in heart failure. Biomedicines. (2024) 12(6):1217. doi: 10.3390/biomedicines12061217, PMID: 38927424 PMC11200601

[122] Du X Dong R Wu Y Ni B . Physiological effects of ferroptosis on organ fibrosis. Oxid Med Cell Longev. (2022) 2022:5295434. doi: 10.1155/2022/5295434, PMID: 36238649 PMC9553398

[123] Sun H Chen D Xin W Ren L Li Q Han X . Targeting ferroptosis as a promising therapeutic strategy to treat cardiomyopathy. Front Pharmacol. (2023) 14:1146651. doi: 10.3389/fphar.2023.1146651, PMID: 37138856 PMC10150641

[124] Bi X Wu X Chen J Li X Lin Y Yu Y . Characterization of ferroptosis-triggered pyroptotic signaling in heart failure. Signal Transduct Target Ther. (2024) 9:257. doi: 10.1038/s41392-024-01962-6, PMID: 39327446 PMC11427671

[125] Liu B Zhao C Li H Chen X Ding Y Xu S . Puerarin Protects against Heart Failure Induced by Pressure Overload through Mitigation of Ferroptosis. Biochem Biophys Res Commun. (2018) 497:233–40. doi: 10.1016/j.bbrc.2018.02.061, PMID: 29427658

[126] Fonseka O Gare SR Chen X Zhang J Alatawi NH Ross C . Molecular mechanisms underlying heart failure and their therapeutic potential. Cells. (2025) 14(5):324. doi: 10.3390/cells14050324, PMID: 40072053 PMC11899429

[127] Zhang Z Tang J Song J Xie M Liu Y Dong Z . Elabela alleviates ferroptosis, myocardial remodeling, fibrosis and heart dysfunction in hypertensive mice by modulating the Il-6/Stat3/Gpx4 signaling. Free Radic Biol Med. (2022) 181:130–42. doi: 10.1016/j.freeradbiomed.2022.01.020, PMID: 35122997

[128] Yang X Kawasaki NK Min J Matsui T Wang F . Ferroptosis in heart failure. J Mol Cell Cardiol. (2022) 173:141–53. doi: 10.1016/j.yjmcc.2022.10.004, PMID: 36273661 PMC11225968

[129] Lopaschuk GD Karwi QG Tian R Wende AR Abel ED . Cardiac energy metabolism in heart failure. Circ Res. (2021) 128:1487–513. doi: 10.1161/circresaha.121.318241, PMID: 33983836 PMC8136750

[130] Cairo G Recalcati S . Iron-regulatory proteins: molecular biology and pathophysiological implications. Expert Rev Mol Med. (2007) 9:1–13. doi: 10.1017/s1462399407000531, PMID: 18053288 PMC2811384

[131] Jiang F Zhang W Lu H Tan M Zeng Z Song Y . Prediction of herbal medicines based on immune cell infiltration and immune- and ferroptosis-related gene expression levels to treat valvular atrial fibrillation. Front Genet. (2022) 13:886860. doi: 10.3389/fgene.2022.886860, PMID: 36246656 PMC9554472

[132] Jin C Zhong Z Gao L Wu X Zhou C Zhou G . Focus on the role of inflammation as a bridge between ferroptosis and atrial fibrillation: A narrative review and novel perspective. Rev Cardiovasc Med. (2024) 25:110. doi: 10.31083/j.rcm2504110, PMID: 39076556 PMC11264012

[133] Yue H Zhan Y Zhang Z Liang W Wu Z . The emerging role of ferroptosis in myocardial fibrosis of atrial fibrillation. Arch Med Sci. (2023) 19:507–12. doi: 10.5114/aoms/160941, PMID: 37034539 PMC10074302

[134] Zhou JB Qian LL Wu D Wang RX . The role of ferroptosis in atrial fibrillation: A promising future. Rev Cardiovasc Med. (2024) 25:127. doi: 10.31083/j.rcm2504127, PMID: 39076535 PMC11264045

[135] Khalid M Petroianu G Adem A . Advanced glycation end products and diabetes mellitus: mechanisms and perspectives. Biomolecules. (2022) 12(4):542. doi: 10.3390/biom12040542, PMID: 35454131 PMC9030615

[136] Zhang W Lu J Wang Y Sun P Gao T Xu N . Canagliflozin attenuates lipotoxicity in cardiomyocytes by inhibiting inflammation and ferroptosis through activating Ampk pathway. Int J Mol Sci. (2023) 24(1):858. doi: 10.3390/ijms24010858, PMID: 36614295 PMC9821072

[137] Guo S Mao X Li X Ouyang H . Association between iron status and incident coronary artery disease: A population based-cohort study. Sci Rep. (2022) 12:17490. doi: 10.1038/s41598-022-22275-0, PMID: 36261681 PMC9581887

[138] Chakraborty A Li Y Zhang C Li Y LeMaire SA Shen YH . Programmed cell death in aortic aneurysm and dissection: A potential therapeutic target. J Mol Cell Cardiol. (2022) 163:67–80. doi: 10.1016/j.yjmcc.2021.09.010, PMID: 34597613 PMC8816882

[139] Shi D Zhang M Zhang Y Shi Y Liu X Wu X . The pathophysiological role of vascular smooth muscle cells in abdominal aortic aneurysm. Cells. (2025) 14(13):1009. doi: 10.3390/cells14131009, PMID: 40643529 PMC12248496

[140] Li B Wang Z Hong J Che Y Chen R Hu Z . Iron deficiency promotes aortic medial degeneration via destructing cytoskeleton of vascular smooth muscle cells. Clin Transl Med. (2021) 11:e276. doi: 10.1002/ctm2.276, PMID: 33463069 PMC7805404

[141] Ji QX Zeng FY Zhou J Wu WB Wang XJ Zhang Z . Ferroptotic stress facilitates smooth muscle cell dedifferentiation in arterial remodelling by disrupting mitochondrial homeostasis. Cell Death Differ. (2023) 30:457–74. doi: 10.1038/s41418-022-01099-5, PMID: 36477078 PMC9950429

[142] Phaelante A Rohde LE Lopes A Olsen V Tobar SA Cohen C . N-acetylcysteine plus deferoxamine improves cardiac function in Wistar rats after non-reperfused acute myocardial infarction. J Cardiovasc Transl Res. (2015) 8:328–37. doi: 10.1007/s12265-015-9633-5, PMID: 26085187

[143] Yan HF Tuo QZ Yin QZ Lei P . The pathological role of ferroptosis in ischemia/reperfusion-related injury. Zool Res. (2020) 41:220–30. doi: 10.24272/j.issn.2095-8137.2020.042, PMID: 32314558 PMC7231469

[144] Yang L Xie P Wu J Yu J Li X Ma H . Deferoxamine treatment combined with sevoflurane postconditioning attenuates myocardial ischemia-reperfusion injury by restoring Hif-1/Bnip3-mediated mitochondrial autophagy in Gk rats. Front Pharmacol. (2020) 11:6. doi: 10.3389/fphar.2020.00006, PMID: 32140105 PMC7042377

[145] Lamichhane A Sharma S Bastola B Chhusyabaga B Shrestha N Poudel P . Unlocking the potential of deferoxamine: A systematic review on its efficacy and safety in alleviating myocardial ischemia-reperfusion injury in adult patients following cardiopulmonary bypass compared to standard care. Ther Adv Cardiovasc Dis. (2024) 18:17539447241277382. doi: 10.1177/17539447241277382, PMID: 39291696 PMC11418332

[146] Ramezanpour M Smith JLP Ooi ML Gouzos M Psaltis AJ Wormald PJ . Deferiprone has anti-inflammatory properties and reduces fibroblast migration *in vitro*. Sci Rep. (2019) 9:2378. doi: 10.1038/s41598-019-38902-2, PMID: 30787349 PMC6382764

[147] Piga A Roggero S Vinciguerra T Sacchetti L Gallo V Longo F . Deferiprone: new insight. Ann N Y Acad Sci. (2005) 1054:169–74. doi: 10.1196/annals.1345.019, PMID: 16339662

[148] Entezari S Haghi SM Norouzkhani N Sahebnazar B Vosoughian F Akbarzadeh D . Iron chelators in treatment of iron overload. J Toxicol. (2022) 2022:4911205. doi: 10.1155/2022/4911205, PMID: 35571382 PMC9098311

[149] Wu YT Zhang GY Hua Y Fan HJ Han X Xu HL . Ferrostatin-1 suppresses cardiomyocyte ferroptosis after myocardial infarction by activating Nrf2 signaling. J Pharm Pharmacol. (2023) 75:1467–77. doi: 10.1093/jpp/rgad080, PMID: 37738327

[150] Mei SL Xia ZY Qiu Z Jia YF Zhou JJ Zhou B . Shenmai injection attenuates myocardial ischemia/reperfusion injury by targeting Nrf2/Gpx4 signalling-mediated ferroptosis. Chin J Integr Med. (2022) 28:983–91. doi: 10.1007/s11655-022-3620-x, PMID: 35997859

[151] Yao D Bao L Wang S Tan M Xu Y Wu T . Isoliquiritigenin alleviates myocardial ischemia-reperfusion injury by regulating the Nrf2/Ho-1/Slc7a11/Gpx4 axis in mice. Free Radic Biol Med. (2024) 221:1–12. doi: 10.1016/j.freeradbiomed.2024.05.012, PMID: 38734270

[152] Cantrell AC Besanson J Williams Q Hoang N Edwards K Bishop GR . Ferrostatin-1 specifically targets mitochondrial iron-sulfur clusters and aconitase to improve cardiac function in sirtuin 3 cardiomyocyte knockout mice. J Mol Cell Cardiol. (2024) 192:36–47. doi: 10.1016/j.yjmcc.2024.05.003, PMID: 38734062 PMC11164624

[153] Xie L Zhao Z Xia H Su S He L Huang Z . A novel tsrna-5008a promotes ferroptosis in cardiomyocytes that causes atrial structural remodeling predisposed to atrial fibrillation. Exp Cell Res. (2024) 435:113923. doi: 10.1016/j.yexcr.2024.113923, PMID: 38190870

[154] Su X Liang F Zeng Y Yang ZR Deng YZ Xu YH . Radiation-induced endothelial ferroptosis accelerates atherosclerosis via the Ddhd2-mediated Nrf2/Gpx4 pathway. Biomolecules. (2024) 14(7):879. doi: 10.3390/biom14070879, PMID: 39062593 PMC11274403

[155] Shen C Wei Y Kang W Wang Q Li G Chen X . Persistent ferroptosis modulates cardiac remodeling and M2 macrophage polarization, which can be mitigated by astaxanthin during myocardial infarction recovery. Cardiovasc Toxicol. (2025) 25:58–73. doi: 10.1007/s12012-024-09942-6, PMID: 39495463

[156] Feng Y Madungwe NB Imam Aliagan AD Tombo N Bopassa JC . Liproxstatin-1 protects the mouse myocardium against ischemia/reperfusion injury by decreasing vdac1 levels and restoring gpx4 levels. Biochem Biophys Res Commun. (2019) 520:606–11. doi: 10.1016/j.bbrc.2019.10.006, PMID: 31623831 PMC7457545

[157] Fang X Ardehali H Min J Wang F . The molecular and metabolic landscape of iron and ferroptosis in cardiovascular disease. Nat Rev Cardiol. (2023) 20:7–23. doi: 10.1038/s41569-022-00735-4, PMID: 35788564 PMC9252571

[158] Wang D Ye P Kong C Chao Y Yu W Jiang X . Mitoferrin 2 deficiency prevents mitochondrial iron overload-induced endothelial injury and alleviates atherosclerosis. Exp Cell Res. (2021) 402:112552. doi: 10.1016/j.yexcr.2021.112552, PMID: 33711329

[159] Nemeth E Ganz T . Hepcidin-ferroportin interaction controls systemic iron homeostasis. Int J Mol Sci. (2021) 22(12):6493. doi: 10.3390/ijms22126493, PMID: 34204327 PMC8235187

[160] Grammer TB Scharnagl H Dressel A Kleber ME Silbernagel G Pilz S . Iron metabolism, hepcidin, and mortality (the ludwigshafen risk and cardiovascular health study). Clin Chem. (2019) 65:849–61. doi: 10.1373/clinchem.2018.297242, PMID: 30917972

[161] Sonkar SK Singh NK Sonkar GK Pandey S Bhosale V Kumar A . Association of hepcidin and anemia in early chronic kidney disease. Saudi J Kidney Dis Transpl. (2019) 30:315–24. doi: 10.4103/1319-2442.256838, PMID: 31031367

[162] Aboelsaad IAF Claggett BL Arthur V Dorbala P Matsushita K Lutsey PL . Hepcidin, incident heart failure and cardiac dysfunction in older adults: the aric study. Eur J Prev Cardiol. (2025) 32(11):993–1000. doi: 10.1093/eurjpc/zwaf018, PMID: 39820727 PMC12264026

[163] Wunderer F Traeger L Sigurslid HH Meybohm P Bloch DB Malhotra R . The role of hepcidin and iron homeostasis in atherosclerosis. Pharmacol Res. (2020) 153:104664. doi: 10.1016/j.phrs.2020.104664, PMID: 31991168 PMC7066581

[164] Liu X Guo Y Huang Y Wang Q Huang Y Lei Y . Gpx4 allosteric activators inhibit ferroptosis and exert myocardial protection in doxorubicin-induced myocardial injury mouse model. Eur J Med Chem. (2024) 277:116721. doi: 10.1016/j.ejmech.2024.116721, PMID: 39096818

[165] Wang X Chen T Chen S Zhang J Cai L Liu C . Sting aggravates ferroptosis-dependent myocardial ischemia-reperfusion injury by targeting gpx4 for autophagic degradation. Signal Transduct Target Ther. (2025) 10:136. doi: 10.1038/s41392-025-02216-9, PMID: 40274801 PMC12022026

[166] Shi Y Zhao Y Sun SJ Lan XT Wu WB Zhang Z . Targeting Gpx4 alleviates ferroptosis and retards abdominal aortic aneurysm formation. Biochem Pharmacol. (2025) 234:116800. doi: 10.1016/j.bcp.2025.116800, PMID: 39952331

[167] Zeng F Chen X Deng G . The anti-ferroptotic role of Fsp1: current molecular mechanism and therapeutic approach. Mol BioMed. (2022) 3:37. doi: 10.1186/s43556-022-00105-z, PMID: 36445538 PMC9708954

[168] Bersuker K Hendricks JM Li Z Magtanong L Ford B Tang PH . The Coq oxidoreductase Fsp1 acts parallel to Gpx4 to inhibit ferroptosis. Nature. (2019) 575:688–92. doi: 10.1038/s41586-019-1705-2, PMID: 31634900 PMC6883167

[169] Doll S Freitas FP Shah R Aldrovandi M Da Silva MC Ingold I . Fsp1 is a glutathione-independent ferroptosis suppressor. Nature. (2019) 575:693–8. doi: 10.1038/s41586-019-1707-0, PMID: 31634899

[170] Hariri E Kassis N Iskandar JP Schurgers LJ Saad A Abdelfattah O . Vitamin K(2)-a neglected player in cardiovascular health: A narrative review. Open Heart. (2021) 8(2):e001015. doi: 10.1136/openhrt-2021-001715, PMID: 34785587 PMC8596038

[171] Qiu C Zheng H Tao H Yu W Jiang X Li A . Vitamin K2 inhibits rat vascular smooth muscle cell calcification by restoring the Gas6/Axl/Akt anti-apoptotic pathway. Mol Cell Biochem. (2017) 433:149–59. doi: 10.1007/s11010-017-3023-z, PMID: 28386842

[172] Hausse AO Aggoun Y Bonnet D Sidi D Munnich A Rötig A . Idebenone and reduced cardiac hypertrophy in Friedreich's Ataxia. Heart. (2002) 87:346–9. doi: 10.1136/heart.87.4.346, PMID: 11907009 PMC1767068

[173] Pan W Zhou G Hu M Li G Zhang M Yang H . Coenzyme Q10 mitigates macrophage mediated inflammation in heart following myocardial infarction via the Nlrp3/Il1β Pathway. BMC Cardiovasc Disord. (2024) 24:76. doi: 10.1186/s12872-024-03729-x, PMID: 38281937 PMC10822151

[174] Al Saadi T Assaf Y Farwati M Turkmani K Al-Mouakeh A Shebli B . Coenzyme Q10 for heart failure. Cochrane Database Syst Rev. (2021) 2):Cd008684. doi: 10.1002/14651858.CD008684.pub3, PMID: 35608922 PMC8092430

[175] Huang A Wang Z Tang H Jia Z Ji X Yang X . Bardoxolone methyl ameliorates myocardial ischemia/reperfusion injury by activating the Nrf2/Ho-1 signaling pathway. Cardiovasc Ther. (2023) 2023:5693732. doi: 10.1155/2023/5693732, PMID: 36874247 PMC9977528

[176] Tian C Gao L Zhang A Hackfort BT Zucker IH . Therapeutic effects of Nrf2 activation by bardoxolone methyl in chronic heart failure. J Pharmacol Exp Ther. (2019) 371:642–51. doi: 10.1124/jpet.119.261792, PMID: 31601682 PMC6849407

[177] McCullough PA Ali S . Cardiac and renal function in patients with type 2 diabetes who have chronic kidney disease: potential effects of bardoxolone methyl. Drug Des Devel Ther. (2012) 6:141–9. doi: 10.2147/dddt.S26714, PMID: 22787387 PMC3392144

[178] Zeng W Wu D Sun Y Suo Y Yu Q Zeng M . The selective Nlrp3 inhibitor Mcc950 hinders atherosclerosis development by attenuating inflammation and pyroptosis in macrophages. Sci Rep. (2021) 11:19305. doi: 10.1038/s41598-021-98437-3, PMID: 34588488 PMC8481539

[179] Zheng G He F Xu J Hu J Ge W Ji X . The selective Nlrp3-inflammasome inhibitor Mcc950 mitigates post-resuscitation myocardial dysfunction and improves survival in a rat model of cardiac arrest and resuscitation. Cardiovasc Drugs Ther. (2023) 37:423–33. doi: 10.1007/s10557-021-07282-z, PMID: 34973094 PMC10164003

[180] Gao R Shi H Chang S Gao Y Li X Lv C . The selective Nlrp3-inflammasome inhibitor Mcc950 reduces myocardial fibrosis and improves cardiac remodeling in a mouse model of myocardial infarction. Int Immunopharmacol. (2019) 74:105575. doi: 10.1016/j.intimp.2019.04.022, PMID: 31299609

[181] Shi Y Zhao L Wang J Liu S Zhang Y Qin Q . The selective Nlrp3 inflammasome inhibitor Mcc950 improves isoproterenol-induced cardiac dysfunction by inhibiting cardiomyocyte senescence. Eur J Pharmacol. (2022) 937:175364. doi: 10.1016/j.ejphar.2022.175364, PMID: 36336012

[182] Aliaga J Bonaventura A Mezzaroma E Dhakal Y Mauro AG Abbate A . Preservation of contractile reserve and diastolic function by inhibiting the Nlrp3 inflammasome with Olt1177(®) (Dapansutrile) in a mouse model of severe ischemic cardiomyopathy due to non-reperfused anterior wall myocardial infarction. Molecules. (2021) 26(12):3534. doi: 10.3390/molecules26123534, PMID: 34207886 PMC8227554

[183] Yang H Zhu J Fu H Shuai W . Dapansutrile ameliorates atrial inflammation and vulnerability to atrial fibrillation in Hfpef rats. Heart Lung Circ. (2024) 33:65–77. doi: 10.1016/j.hlc.2023.09.017, PMID: 38040503

[184] Helseth R Kleveland O Ueland T Wiseth R Damas JK Broch K . Tocilizumab increases citrullinated histone 3 in non-St segment elevation myocardial infarction. Open Heart. (2021) 8(1):e001492. doi: 10.1136/openhrt-2020-001492, PMID: 33972404 PMC8112443

[185] Broch K Anstensrud AK Woxholt S Sharma K Tøllefsen IM Bendz B . Randomized trial of interleukin-6 receptor inhibition in patients with acute St-segment elevation myocardial infarction. J Am Coll Cardiol. (2021) 77:1845–55. doi: 10.1016/j.jacc.2021.02.049, PMID: 33858620

[186] Pottebaum AA January SE Liu C Lavine S Schilling JD Lavine KJ . Feasibility of interleukin-6 receptor blockade in cardiac antibody-mediated rejection. Transplantation. (2024) 108:539–44. doi: 10.1097/tp.0000000000004784, PMID: 37638881 PMC10798586

[187] Woxholt S Ueland T Aukrust P Anstensrud AK Broch K Tøllefsen IM . Cytokine pattern in patients with St-elevation myocardial infarction treated with the interleukin-6 receptor antagonist tocilizumab. Open Heart. (2023) 10(2):e002301. doi: 10.1136/openhrt-2023-002301, PMID: 37591633 PMC10441101

[188] Ye J Lyu TJ Li LY Liu Y Zhang H Wang X . Ginsenoside re attenuates myocardial ischemia/reperfusion induced ferroptosis via Mir-144-3p/Slc7a11. Phytomedicine. (2023) 113:154681. doi: 10.1016/j.phymed.2023.154681, PMID: 36893674

[189] Zhong G Chen J Li Y Han Y Wang M Nie Q . Ginsenoside Rg3 attenuates myocardial ischemia/reperfusion-induced ferroptosis via the Keap1/Nrf2/Gpx4 signaling pathway. BMC Complement Med Ther. (2024) 24:247. doi: 10.1186/s12906-024-04492-4, PMID: 38926825 PMC11209975

[190] Zhai Y Bai J Peng Y Cao J Fang G Dong Y . Ginsenoside Rb1 attenuates doxorubicin induced cardiotoxicity by suppressing autophagy and ferroptosis. Biochem Biophys Res Commun. (2024) 710:149910. doi: 10.1016/j.bbrc.2024.149910, PMID: 38593619

[191] Xu X Wu Q Pei K Zhang M Mao C Zhong X . Ginsenoside Rg1 reduces cardiac inflammation against myocardial ischemia/reperfusion injury by inhibiting macrophage polarization. J Ginseng Res. (2024) 48:570–80. doi: 10.1016/j.jgr.2024.07.003, PMID: 39583164 PMC11583468

[192] Yu P Li Y Fu W Li X Liu Y Wang Y . Saponins protect myocardial ischemia reperfusion no-reflow through inhibiting the activation of Nlrp3 inflammasome via Tlr4/Myd88/Nf-Kb signaling pathway. Front Pharmacol. (2020) 11:607813. doi: 10.3389/fphar.2020.607813, PMID: 33628178 PMC7898550

[193] Liu J Pei C Jia N Han Y Zhao S Shen Z . Preconditioning with ginsenoside Rg3 mitigates cardiac injury induced by high-altitude hypobaric hypoxia exposure in mice by suppressing ferroptosis through inhibition of the rhoa/rock signaling pathway. J Ethnopharmacol. (2025) 337:118861. doi: 10.1016/j.jep.2024.118861, PMID: 39326813

[194] Xu H Pang M Hu C Xu T Chen G Zhang G . Panax ginseng exerts cardioprotective effect post myocardial infarction by attenuating myocardial fibrosis and inflammation through Sirt1 signaling pathways. J Ginseng Res. (2025) 49:197–207. doi: 10.1016/j.jgr.2025.01.001, PMID: 40061482 PMC11889384

[195] Li J Li Y Yuan X Yao D Gao Z Niu Z . The effective constituent puerarin, from pueraria lobata, inhibits the proliferation and inflammation of vascular smooth muscle in atherosclerosis through the Mir-29b-3p/Igf1 pathway. Pharm Biol. (2023) 61:1–11. doi: 10.1080/13880209.2022.2099430, PMID: 36537316 PMC9788726

[196] Li X Yuan T Chen D Chen Y Sun S Wang D . Cardioprotective effects of puerarin-V on isoproterenol-induced myocardial infarction mice is associated with regulation of Ppar-Υ/Nf-Kb pathway. Molecules. (2018) 23(12):3322. doi: 10.3390/molecules23123322, PMID: 30558188 PMC6321361

[197] Hu F Hu T He A Yuan Y Wang X Zou C . Puerarin protects myocardium from ischaemia/reperfusion injury by inhibiting ferroptosis through downregulation of Vdac1. J Cell Mol Med. (2024) 28:e70313. doi: 10.1111/jcmm.70313, PMID: 39730981 PMC11680193

[198] Wang ZK Chen RR Li JH Chen JY Li W Niu XL . Puerarin protects against myocardial ischemia/reperfusion injury by inhibiting inflammation and the Nlrp3 inflammasome: the role of the Sirt1/Nf-Kb pathway. Int Immunopharmacol. (2020) 89:107086. doi: 10.1016/j.intimp.2020.107086, PMID: 33068868

[199] Zhou B Zhang J Chen Y Liu Y Tang X Xia P . Puerarin Protects against Sepsis-Induced Myocardial Injury through Ampk-Mediated Ferroptosis Signaling. Aging (Albany NY). (2022) 14:3617–32. doi: 10.18632/aging.204033, PMID: 35482440 PMC9085223

[200] Fontana F Molinaro G Moroni S Pallozzi G Ferreira MPA Tello RP . Biomimetic platelet-cloaked nanoparticles for the delivery of anti-inflammatory curcumin in the treatment of atherosclerosis. Adv Healthc Mater. (2024) 13:e2302074. doi: 10.1002/adhm.202302074, PMID: 38499190 PMC11468963

[201] Chen FY Zhou J Guo N Ma WG Huang X Wang H . Curcumin retunes cholesterol transport homeostasis and inflammation response in M1 macrophage to prevent atherosclerosis. Biochem Biophys Res Commun. (2015) 467:872–8. doi: 10.1016/j.bbrc.2015.10.051, PMID: 26471308

[202] Momtazi-Borojeni AA Abdollahi E Nikfar B Chaichian S Ekhlasi-Hundrieser M . Curcumin as a potential modulator of M1 and M2 macrophages: new insights in atherosclerosis therapy. Heart Fail Rev. (2019) 24:399–409. doi: 10.1007/s10741-018-09764-z, PMID: 30673930

[203] Rahnavard M Hassanpour M Ahmadi M Heidarzadeh M Amini H Javanmard MZ . Curcumin ameliorated myocardial infarction by inhibition of cardiotoxicity in the rat model. J Cell Biochem. (2019) 120:11965–72. doi: 10.1002/jcb.28480, PMID: 30775806

[204] Chen TH Yang YC Wang JC Wang JJ . Curcumin treatment protects against renal ischemia and reperfusion injury-induced cardiac dysfunction and myocardial injury. Transplant Proc. (2013) 45:3546–9. doi: 10.1016/j.transproceed.2013.09.006, PMID: 24314955

[205] Yuan Y Huang H Hu T Zou C Qiao Y Fang M . Curcumin pretreatment attenuates myocardial ischemia/reperfusion injury by inhibiting ferroptosis, autophagy and apoptosis via Hes1. Int J Mol Med. (2024) 54(6):110. doi: 10.3892/ijmm.2024.5434, PMID: 39364745 PMC11517743

[206] Jiang C Shi Q Yang J Ren H Zhang L Chen S . Ceria nanozyme coordination with curcumin for treatment of sepsis-induced cardiac injury by inhibiting ferroptosis and inflammation. J Adv Res. (2024) 63:159–70. doi: 10.1016/j.jare.2023.10.011, PMID: 37871772 PMC11380017

[207] Li X Fang Q Tian X Wang X Ao Q Hou W . Curcumin attenuates the development of thoracic aortic aneurysm by inhibiting Vegf expression and inflammation. Mol Med Rep. (2017) 16:4455–62. doi: 10.3892/mmr.2017.7169, PMID: 28791384 PMC5647005

[208] Yang Y Hong M Lian WW Chen Z . Review of the pharmacological effects of astragaloside iv and its autophagic mechanism in association with inflammation. World J Clin cases. (2022) 10:10004–16. doi: 10.12998/wjcc.v10.i28.10004, PMID: 36246793 PMC9561601

[209] Kang X Su S Hong W Geng W Tang H . Research progress on the ability of astragaloside iv to protect the brain against ischemia-reperfusion injury. Front Neurosci. (2021) 15:755902. doi: 10.3389/fnins.2021.755902, PMID: 34867166 PMC8637115

[210] Tan YQ Chen HW Li J . Astragaloside iv: an effective drug for the treatment of cardiovascular diseases. Drug Des Devel Ther. (2020) 14:3731–46. doi: 10.2147/dddt.S272355, PMID: 32982178 PMC7507407

[211] Meng P Yang R Jiang F Guo J Lu X Yang T . Molecular mechanism of astragaloside iv in improving endothelial dysfunction of cardiovascular diseases mediated by oxidative stress. Oxid Med Cell Longev. (2021) 2021:1481236. doi: 10.1155/2021/1481236, PMID: 34840664 PMC8626190

[212] Zhang Y Du M Wang J Liu P . Astragaloside iv relieves atherosclerosis and hepatic steatosis via Mapk/Nf-Kb signaling pathway in Ldlr(-/-) mice. Front Pharmacol. (2022) 13:828161. doi: 10.3389/fphar.2022.828161, PMID: 35264962 PMC8899310

[213] Shi H Zhou P Gao G Liu PP Wang SS Song R . Astragaloside iv prevents acute myocardial infarction by inhibiting the Tlr4/Myd88/Nf-Kb signaling pathway. J Food Biochem. (2021) 45:e13757. doi: 10.1111/jfbc.13757, PMID: 34032295

[214] Zhai P Chen Q Wang X Ouyang X Yang M Dong Y . The combination of tanshinone iia and astragaloside iv attenuates myocardial ischemia-reperfusion injury by inhibiting the sting pathway. Chin Med. (2024) 19:34. doi: 10.1186/s13020-024-00908-y, PMID: 38419127 PMC10900662

[215] Wang X Chen X Wang Y He X Li L Wang X . Astragaloside iv alleviates inflammation and improves myocardial metabolism in heart failure mice with preserved ejection fraction. Front Pharmacol. (2024) 15:1467132. doi: 10.3389/fphar.2024.1467132, PMID: 39640484 PMC11618538

[216] Li X Li Z Dong X Wu Y Li B Kuang B . Astragaloside iv attenuates myocardial dysfunction in diabetic cardiomyopathy rats through downregulation of Cd36-mediated ferroptosis. Phytother Res. (2023) 37:3042–56. doi: 10.1002/ptr.7798, PMID: 36882189

[217] Wang J Zhou Y Wu S Huang K Thapa S Tao L . Astragaloside iv attenuated 3,4-benzopyrene-induced abdominal aortic aneurysm by ameliorating macrophage-mediated inflammation. Front Pharmacol. (2018) 9:496. doi: 10.3389/fphar.2018.00496, PMID: 29872394 PMC5972279

[218] Yang A Zhang H Zhang H Li N Chen C Yang X . Pitavastatin and resveratrol bio-nanocomplexes against hyperhomocysteinemia-induced atherosclerosis via blocking ferroptosis-related lipid deposition. J Control Release. (2025) 381:113598. doi: 10.1016/j.jconrel.2025.113598, PMID: 40043912

[219] Liu J Zhang M Qin C Wang Z Chen J Wang R . Resveratrol attenuate myocardial injury by inhibiting ferroptosis via inducing Kat5/Gpx4 in myocardial infarction. Front Pharmacol. (2022) 13:906073. doi: 10.3389/fphar.2022.906073, PMID: 35685642 PMC9171715

[220] Li T Tan Y Ouyang S He J Liu L . Resveratrol protects against myocardial ischemia-reperfusion injury via attenuating ferroptosis. Gene. (2022) 808:145968. doi: 10.1016/j.gene.2021.145968, PMID: 34530090

[221] Hu T Zou HX Zhang ZY Wang YC Hu FJ Huang WX . Resveratrol protects cardiomyocytes against ischemia/reperfusion-induced ferroptosis via inhibition of the Vdac1/Gpx4 pathway. Eur J Pharmacol. (2024) 971:176524. doi: 10.1016/j.ejphar.2024.176524, PMID: 38561102

[222] Zhang W Qian S Tang B Kang P Zhang H Shi C . Resveratrol inhibits ferroptosis and decelerates heart failure progression via Sirt1/P53 pathway activation. J Cell Mol Med. (2023) 27:3075–89. doi: 10.1111/jcmm.17874, PMID: 37487007 PMC10568670

[223] Vasamsetti SB Karnewar S Gopoju R Gollavilli PN Narra SR Kumar JM . Resveratrol attenuates monocyte-to-macrophage differentiation and associated inflammation via modulation of intracellular Gsh homeostasis: relevance in atherosclerosis. Free Radic Biol Med. (2016) 96:392–405. doi: 10.1016/j.freeradbiomed.2016.05.003, PMID: 27156686

[224] Li J Xie C Zhuang J Li H Yao Y Shao C . Resveratrol attenuates inflammation in the rat heart subjected to ischemia-reperfusion: role of the Tlr4/Nf-Kb signaling pathway. Mol Med Rep. (2015) 11:1120–6. doi: 10.3892/mmr.2014.2955, PMID: 25405531

[225] Gal R Deres L Horvath O Eros K Sandor B Urban P . Resveratrol improves heart function by moderating inflammatory processes in patients with systolic heart failure. Antioxidants (Basel). (2020) 9(11):1108. doi: 10.3390/antiox9111108, PMID: 33187089 PMC7696241

[226] Qu Y Zhang N Zhao Y . Resveratrol inhibits abdominal aortic aneurysm progression by reducing extracellular matrix degradation, apoptosis, autophagy, and inflammation of vascular smooth muscle cells via upregulation of Hmox1. J Endovasc Ther. (2025) 32:1224–36. doi: 10.1177/15266028231202727, PMID: 37789605

[227] Cruz-Gregorio A Amezcua-Guerra LM Fisher-Bautista B Romero-Beltrán A Fonseca-Camarillo G . The protective role of interleukin-37 in cardiovascular diseases through ferroptosis modulation. Int J Mol Sci. (2024) 25(18):9758. doi: 10.3390/ijms25189758, PMID: 39337246 PMC11432013

[228] Behrens F Bartolomaeus H Wilck N Holle J . Gut-immune axis and cardiovascular risk in chronic kidney disease. Clin Kidney J. (2024) 17:sfad303. doi: 10.1093/ckj/sfad303, PMID: 38229879 PMC10790347

[229] Zhou W Thiery JP . Ferroptosis-related Lncrnas in diseases. BMC Biol. (2025) 23:158. doi: 10.1186/s12915-025-02268-x, PMID: 40481573 PMC12143037

